# Helminth infections among rural schoolchildren in Southern Ethiopia: A cross-sectional multilevel and zero-inflated regression model

**DOI:** 10.1371/journal.pntd.0008002

**Published:** 2020-12-22

**Authors:** Hiwot Hailu Amare, Bernt Lindtjørn

**Affiliations:** 1 School of Public Health, College of Medicine and Health Sciences, Hawassa University, Hawassa, Ethiopia; 2 Centre for International Health, University of Bergen, Bergen, Norway; 3 Department of Public Health, College of Health Sciences and Medicine, Dilla University, Dilla, Ethiopia; University of Pennsylvania School of Veterinary Medicine, UNITED STATES

## Abstract

Although the prevalence of helminths infection among schoolchildren is known, there has been little progress in the application of count model for modelling the risk factors of helminths egg. Only a few studies applied multilevel analysis to explore the variation in helminths prevalence across schools and classes. This study aimed to assess the prevalence, intensity of helminths infection, and identify risk factors at the individual-, household-, and school-level among schoolchildren in Southern Ethiopia. Using multistage random sampling, we recruited 864 students in the Wonago District. We applied multilevel-logistic and zero-inflated negative binomial regression models (ZINB). Risk factors were concentrated at the individual level; school-level and class-level variables explained less than 5% of the variance. The overall helminths prevalence was 56% (479/850); *Trichuris trichiura* prevalence was 42.4% (360/850); and *Ascaris lumbricoides* prevalence was 18.7% (159/850). The rate of any helminths increased among thin children (AOR: 1.73 [95% CI: (1.04, 2.90]), anemic (AOR: 1.45 [95% CI: 1.04, 2.03]), mothers who had no formal education (AOR: 2.08 [95% CI: 1.25, 3.47]), and those in households using open containers for water storage (AOR: 2.06 [95% CI: 1.07, 3.99]). In the ZINB model, *A*. *lumbricoides* infection intensity increased with increasing age (AOR: 1.08 [95% CI: 1.01, 1.16]) and unclean fingernails (AOR: 1.47 [95% CI: 1.07, 2.03]). Handwashing with soap (AOR: 0.68 [95% CI: 0.48, 0.95]), de-worming treatment [AOR: 0.57 (95% CI: 0.33, 0.98)], and using water from protected sources [AOR: 0.46 (95% CI: 0.28, 0.77)] were found to be protective against helminths infection. After controlling for clustering effects at the school and class levels and accounting for excess zeros in fecal egg counts, we found an association between helminths infection and the following variables: age, thinness, anemia, unclean fingernails, handwashing, de-worming treatment, mother’s education, household water source, and water storage protection. Improving hygiene behavior, providing safe water at school and home, and strengthening de-worming programs is required to improve the health of schoolchildren in rural Gedeo.

## Author summary

Helminth infections are common among school-aged children in Ethiopia. Several cross-sectional studies have investigated the risk factors of these helminths infection. However, most were conducted in an urban setting in Northern Ethiopia. Many of these studies did not report the intensity of helminth infections; and they restricted helminths infection data to binary outcomes. There has been limited report related to zero-inflated model for helminths count data with excess zeros and over-dispersion. Multilevel analysis for nested structure of school data has also been rarely applied. Therefore, we aimed to assess the prevalence and intensity of helminths infection and the related individual-, household-, and school-level risk factors among rural schoolchildren in Southern Ethiopia. Using count model, we modelled the risk factors of helminths egg. Using a multivariate, multilevel, mixed-effect, logistic regression model, we found minimal variation across class- and school-level factors for helminths infection prevalence. We found associations between helminths infection and most individual-, and some household-level factors. Therefore, interventions focusing on the individual, household, and school should be implemented to reduce the prevalence of infection and worm load among schoolchildren.

## Introduction

More than 1.5 billion people around the world are infected by soil-transmitted helminths (STHs), including over 568 million schoolchildren who are at risk [1]. In 2015, an approximately 88 million individuals, including 28 million school-aged children, were at risk for STH infections in Ethiopia [2]. Roundworm (*Ascaris lumbricoides)*, whipworm (*Trichuris trichiura*), and hookworm (*Ancylostoma duodenale* and *Necator americanus*) are the most common STH infections that chronically infect children [3]. There were an estimated 5.19 million disability adjusted life years (DALYs) attributable to these infections [4]. The health burdens of STH infections is mainly attributed to their chronic and insidious impact on the health and quality of life because morbidity is considerably high in heavy infection intensity rather than the absence or presence of infection [3, 5, 6]. Loss of appetite, malabsorption, anemia, impaired children’s nutrition, poor physical and intellectual development, and impaired cognitive function can occur with these infections [6, 7]. Some of the risk factors of these infections include poverty [8], mothers’ education, untrimmed fingernails, walking barefoot, unsanitary toilet areas, not washing hands before eating or after visiting the toilet, eating raw or undercooked vegetables or meat, lack of hygiene facilities, and drinking water from unsafe sources [5, 9]. In developing countries, control measures can be difficult to implement due to water and sanitation problems [10].

In Ethiopia, the prevalence of helminths infection among schoolchildren ranges between 18% and 63% [8, 11–17], with the highest rate of infection (63%) recorded in the Southern region [17]. The government of Ethiopia is expanding schooling to make education more relevant to all children and meet their nutritional and health needs [18]. This strategy includes facilitating and implementing a de-worming service every six months and improving water, hygiene, and sanitation facilities [19]. However, many school-aged children continue to be affected by helminthic infections [20–22]. Moreover, most schools have no handwashing facilities, and hygienic behavior is inadequate [23]. Evidence of open defecation is observed in 53% of schools in Southern Ethiopia [24].

Previous studies from Ethiopia are mainly from urban areas in Northern Ethiopia [8, 11–17]. Only a few studies have assessed the prevalence and intensity of helminths infection among schoolchildren in Southern Ethiopia [12, 17, 24], and even fewer from rural areas. Many of previous studies did not report the risk factors of helminths intensity; most interpreted the helminths infection data in terms of binary outcomes. Despite various studies on helminths infection prevalence, there has been little progress in the application of count model for modelling the risk factors of helminths egg concentration in the stool specimen. However, the data related with helminthic egg counts are usually over-dispersed and zero-inflated [25]. Interpretation of such data can be problematic, as these data require the use of specific statistical models during the analysis process. To the best of our knowledge, no study has considered the two generating process for excess zeros and over-dispersion in the distribution of helminths egg count among schoolchildren in Ethiopia. Furthermore, most previous studies did not considered the nested structure of school data (i.e., individuals nested within the same class and classes nested within the same school) in their analysis. This paper is part of a larger study, which aimed to identify school health problems such as anemia, and stunting co-existence and helminth infections and skin problems and the risk factors associated with these problems in the Gedeo area in Southern Ethiopia. Therefore, this paper aimed to report the prevalence and intensity of helminths infection and identifies potential risk factors at the individual-, household-, and school-level among rural schoolchildren in the Wonago district of Southern Ethiopia. Using a multivariate, multilevel, regression model, we identified factors contributing to variations in the prevalence of helminths infection in this population. We also identified factors related with helminths egg intensity using zero-inflated count model.

## Methods

### Ethics statement

The institutional review board at the College of Medicine and Health Sciences of Hawassa University (IRB/005/09) and the Regional Ethical Committee of Western Norway (2016/1900/REK vest) provided ethical clearance. The Gedeo Zone Health Department and District Education Office provided a letter of permission. School directors and teachers participated in discussions. We obtained informed written (signed) and verbal (thumb print) consent from study participants’ parents or guardians and permission (assent) from children aged 12 years and older before the interviews. The participants’ privacy and confidentiality were maintained. Children diagnosed as anemic and who tested positive for helminths infections were referred to the nearest health institution for treatment according to the standard national guidelines [26].

### Study area, design, and participants

The study was conducted in the Wonago district of the Gedeo zone in the Southern Ethiopia. The district is 377 km south of Addis Ababa, the capital city of Ethiopia, and 13 km South of Dilla, the capital city of the Gedeo zone. The district has 17 rural and 4 urban *kebeles*, which is the smallest administrative units. In 2014, Wonago’s population was estimated to be 143,989 people: 71,663 (49.8%) men and 72,326 (50.2%) women. The district is among the most densely populated areas in Ethiopia, with 1,014 people per square kilometre of land area. The district has 26 government health facilities (6 health centers and 20 health posts), 2 private clinics, and 2 drug stores, and more than 36,000 students in 3 urban and 22 rural primary schools. Most residents depend on cash crops of coffee, fruit, and *ensete* (*Ensete ventricosum)*.

**Fig 1:**
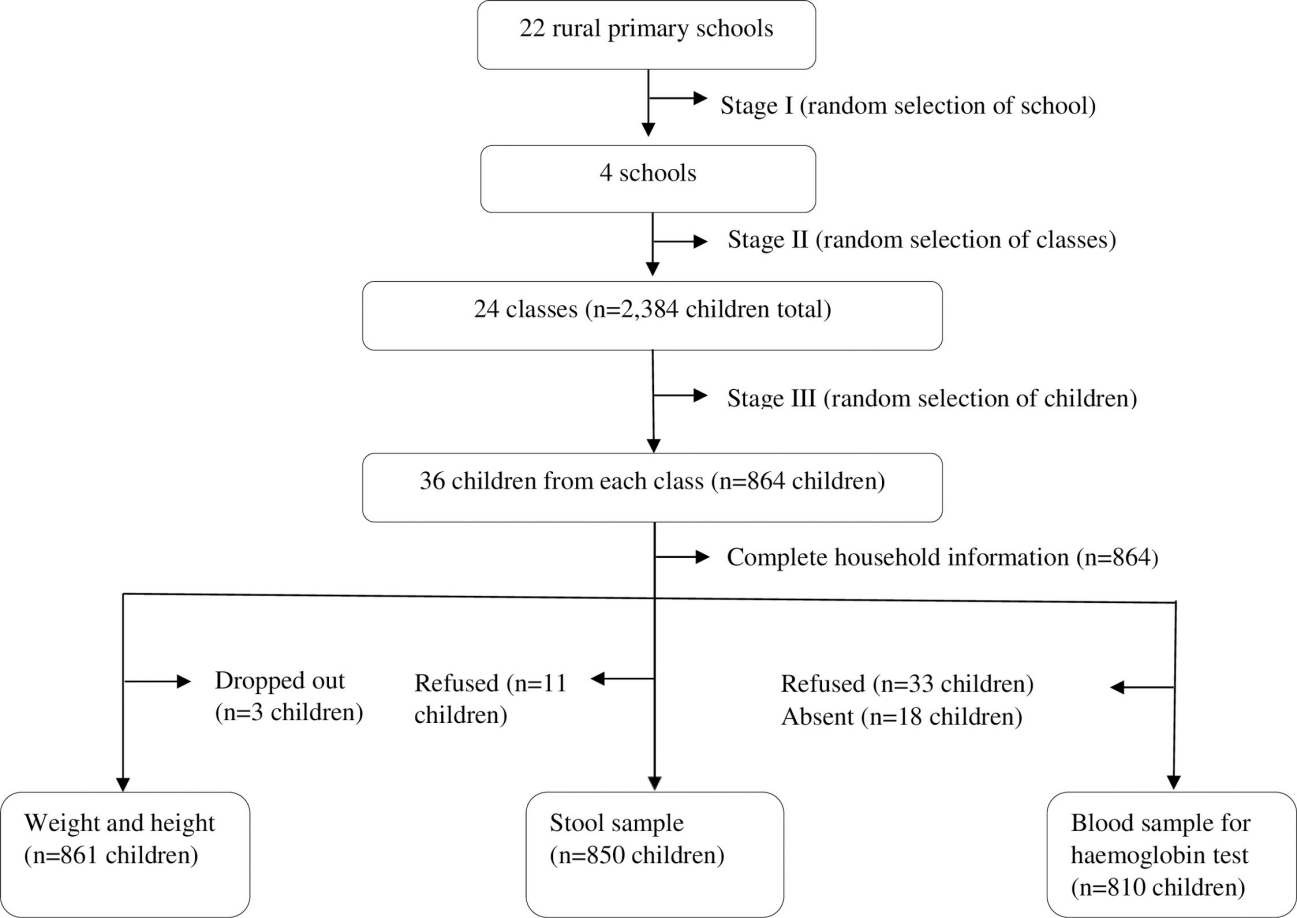
Flowchart of study inclusion.

We conducted this cross-sectional survey from February 2017 to June 2017. The study population was schoolchildren and their parents or guardians. Students aged 7–14 years were recruited in schools and their parents or guardian contacted by visiting their homes. Using a three-stage cluster sampling method, we randomly assigned 4 schools to level one, 24 classes (comprising 2,384) to level two, and 864 students to level three. We then randomly included 36 children from each class. When more than one child in a class was living together in the same household, one of them was selected randomly by a lottery method. The household of the student's parents or guardian was identified through a local guide. The study participants’ parents or guardians consented and children assented before enrolment. We replaced participants who dropped out of school after the selection process with participants of the same class, sex, and age. The recruitment process is shown in Fig 1.

### Sample size

Since this study was part of a large project aiming to identify school health problems, we considered multiple factors to calculate the sample size using OpenEpi software [27] based on single population proportion [28]. Assuming a 95% confidence interval (CI), the maximum sample size was calculated using proportions of different variables from previous studies (e.g., anemia [27%], stunting [30%], thinness [37%], helminths infection [27%], and skin infection [50%]), 5% precision, and a design effect of 2 to account for multistage sampling [12, 29–32]. We also calculated the sample size using outcome-associated variables, such as under-nutrition (32% prevalence of stunting among female participants), and helminths infection (50% among children who did not wash their hands before meals) [12, 29–32]. Finally, we obtained the maximum sample size using 50% of helminths infection among children who did not wash their hands before meals, and 50% of skin infection. The reason that we included skin infection in the sample size calculation was that this problem was one of our studies planned for a subsequent paper. After adding a 10% non-response rate, we reached a final sample size of 845, the minimum required sample size. We then randomly recruited 864 students.

### Data collection tools and procedures

Ten trained enumerators conducted the interviews using a pretested, structured questionnaire that was adapted and developed in English and then translated into the local *Gedeooffa* language. The interviews were done with children at their schools and with parent at their homes. Training was provided for all personnel participated in data collection, supervision, and data entry process. To minimize potential bias and validate the measurement tools prior to actual data collection, a pre-test was conducted on 42 primary school-aged children in other schools not selected for this study. The supervisors checked data for completeness and consistency onsite.

We assessed individual, parent, household, and school level exposure variables. Individual and household factors were collected from the child or the child’s parents or guardians via interviews and observations of the housing conditions. The individual child factors included sex, age, hygiene behavior, loss of appetite in the past month, de-worming treatment in the past 6 months. Measurements such as weight, height, and haemoglobin were done for children in the school. To measure weight, a digital portable scale (Seca 877, Seca GmbH, Germany) was calibrated to the nearest 0.1 kg. Children were weighed in light clothing and no shoes. To measure height, a measuring board (Seca 213, Seca GmbH, Germany) was calibrated to the nearest 0.1 cm. Children were measured while standing barefoot with parallel feet, heels, buttocks, and shoulders, with their heads held upright, the backs of their heads touching the measuring board, and their hands hanging by their sides. Capillary blood samples were taken for haemoglobin measurement, processed, and examined using standard procedures by trained and experienced laboratory technicians using a HemoCue Analyser Hb 301 (Angelholm, Sweden). Parent factors included the educational level of the mother and father. Household factors included the wealth index, which was constructed using principal component analysis of 14 household assets (electricity, radio, television, mobile phone, table, chair, bed, separate kitchen, cooking place, own land, bank account, toilet facility, floor type, and roof type); family size; source of drinking water; container used to store water; and use of treated water. School factors included access to health education on personal hygiene, absence from school in the past month, and participation in the school food program.

### Laboratory procedures

Stool samples were collected, processed, and examined using standard procedures [33, 34]. Samples were collected at school in the early morning and stored in stool cups labelled with an identification code, name, sex, age, and date. The specimens were transported in a cold-box with frozen ice-packs to the nearest health facility, Dilla University Teaching and Referral Hospital, where Kato-Katz and formalin-ether concentration (FEC) techniques were used to conduct stool tests. Single, 41.7-mg thick, Kato-Katz smears were prepared from each stool sample on the same day of specimen collection. Then, 1 g of stool was preserved in 10% formalin solution and processed using the FEC technique [35]. All Kato-Katz slides were examined within one hour of preparation to minimize the risk of hookworm egg disappearance, and then the reading of slides were re-performed to detect and count egg of other helminth infections. The slides were examined by three experienced laboratory technicians. The result of each helminths species from two diagnostic techniques was recorded separately. To reduce possible bias introduced during outcome measurement, 10% of 850 of the test results of Kato-Katz and FEC were randomly selected and re-examined in a blinded fashion to ensure reproducibility of the results. To ensure accuracy of helminth results, the quality control was performed based on World Health Organization (WHO) guideline [36]. According to WHO guideline, re-reading is required if the expert identifies a difference in the egg count of more than 10% and more than four eggs between the readings and discuss the reasons for the discrepancy [36]. However, in the WHO guideline, there is no clear information how to handle differences in presence or absence of helminth eggs [37]. Therefore, we compared the helminths egg count of initial reading with second quality control reading. According to WHO guideline, when the difference in helminths egg count exceed four eggs, re-reading of slides was performed by the third senior laboratory technician. In case of a discordant results between the initial reading and re-reading were confirmed by the third senior laboratory technician. However, the WHO quality control guideline is mainly for quantitative diagnostic methods such as Kato-Katz method, there is no guideline for judging discrepancy of faecal egg counts obtained by the FEC semi-quantitative method for quality control. Hence, we performed the quality control for the FEC method based on the presence or absence of helminth eggs. For the FEC method, a discordant result was considered when there is difference in the presence or absence helminths egg from initial reading to the quality control reading. Results were classified as false-positive if the original result was positive for a specific helminths infection, but the results from the second reading, as well as from the third reading, were negative. Results were classified as false-negative if the original result was negative, but the quality control as well as the result from the third reading, were positive.

### Statistical analysis

The data were entered into a database using the double-entry system in Epi-data version 3.1 (EpiData Association; Odense Denmark, 2004). Inconsistencies were cleaned and missing values addressed before analysis. After validation, the data were exported to SPSS version 20 (IBM Corp, 2011) and STATA 14 software (StataCorp LP, College Station, TX, 2015) for analysis.

Descriptive statistics including frequency, percentage, mean, median, range, interquartile range (IQR), and standard deviation (SD) were calculated to describe relevant variables. Cross tabulation was used to calculate the proportions of categorical variables in relation to outcome variables for any helminths infection, for *T*. *trichiura* infection, and for *A*. *lumbricoides* infection. A wealth index was constructed by using principal component analysis [38] to code the previously listed 14 household assets as 0 (absent) or 1 (present). The internal consistency of the 14 variables was determined (Cronbach alpha of 0.78 and Kaiser-Meyer-Olkin sampling adequacy of 0.8). The socioeconomic indictors (poor, middle, and rich) were categorized based on the first component explaining 28.3% of the variance in the data with an Eigen value of 4.1. We used WHO AnthroPlus 1.0.4 software to calculate height-for-age, weight-for-age, and body mass index-for-age Z scores according to the standard reference for children aged 5–19 year [39]. Stunting was defined as height-for-age Z scores < -2 SD, and thinness was defined as body-mass-index-for-age Z scores < -2 SD [40]. Anaemia was recorded according to the WHO guidelines for school-aged children: haemoglobin < 11.5 g/dl for those aged 5–11 years and < 12 g/dl for those aged 12–14 years. Anaemia was estimated using the haemoglobin value adjusted for altitude [41].

Kappa statistics were used to estimate reliability of the inter-rater agreement of the two readers using 10% of the test results in either Kato-Katz or FEC method. Kappa values were defined as follows: poor = 0.01–0.2; fair = 0.21–0.4; moderate = 0.41–0.6; good = 0.61–0.8; and perfect = 0.81–1 [42]. Kappa values were considered statistically at P < 0.05.

We used a multivariate, multilevel, mixed-effect, logistic regression model to analyze three separate binary outcome variables: the presence or absence of any helminth infections, the presence or absence of *T*. *trichiura*, and the presence or absence of *A*. *lumbricoides*. Any helminth infections in this study includes (*T*. *trichiura*, *A*. *lumbricoides*, *Taenia* species, hookworm species, *Strongyloides stercoralis*, and *Hymenolepis nana*). In addition, the eggs detected per Kato-Katz slide (41.7 mg of faeces) was multiplied by a factor 24 to obtain a standard measure of eggs per gram (epg) of stool [43]. The epg was then used as a proxy for estimation of helminths infection intensity. Infection intensity was defined as light (< 5,000 epg for *A*. *lumbricoides;* < 1,000 epg for *T*. *trichiura;* and < 2,000 epg for hookworm) or moderate (≥ 5,000 epg for *A*. *lumbricoides*; ≥ 1,000 epg for *T*. *trichiura*; and ≥ 2,000 epg for hookworm) [44]. Two separate count models also were constructed for the *T*. *trichiura* and *A*. *lumbricoides* fecal egg counts. Thus, using a zero-inflated negative binomial (ZINB) regression model, we performed two part model; the first part has an interpretation as binary outcome model and the second part as a count model. The association between the over-dispersed count outcome with excess zeros and the potential predictors of *T*. *trichiura* and *A*. *lumbricoides* infection was determined using a ZINB regression model [25].

### Multilevel, mixed-effect, logistic regression for modelling infection risk

We used three data hierarchies: school-level, class-level, and individual-level (child or parent). Student participants were clustered within the same class, and classes were nested within schools. We included school and class levels during the analysis and assessed potential confounding and effect modifications using a multivariate, multilevel, mixed-effect, logistic regression model and stratified analysis. Prior to the multivariate regression, we checked the collinearity among exposure variables. We used the presence and absence of any helminths, of *T*. *trichiura* infection, and of *A*. *lumbricoides* infection as separate outcome variables and conducted the analysis using a multilevel logistic regression model. For all predictors, we applied a simple, bivariate, logistic regression without considering random effects, and a multilevel, logistic regression model with random school and class effects. We also estimated the intra-cluster correlation coefficient for each model within school and class.

Five models were constructed for each outcome variable (any helminths or *T*. *trichiura*, or *A*. *lumbricoides* infection). Model 1 (empty) had no covariate indicating whether to consider the random-effect model. Model II contained the individual child factors. Model III contained the individual child and individual parent factors. Model IV contained household, individual child, and parent factors. Finally, Model V used multilevel, multivariate, logistic regression to assess individual, household, and school factors. Exposure variables with P values < .25 in the bivariate multilevel logistic regression model were introduced into the model II, model III and model IV.

Variables in model V for any helminths included individual child factors (sex, age, loss of appetite in past month, nail trimming, handwashing with soap before meals, thinness, and anemia), individual parent factors (mother’s educational status), household factors (wealth, source of drinking water, container used to store water, treated water), and school factors (participation in school feeding program). Covariate variables (e.g., sex, age, nail trimming, handwashing with soap before meals, wealth, source of drinking water, using treated water, and participation in a school feeding program) with P values >.25 in the bivariate regression model were retained in the final model to control for confounding.

Variables in model V for *T*. *trichiura* infection included individual child factors (sex, age, loss of appetite in past month, eating uncooked vegetable, thinness, and anemia), individual parent factors (mother’s educational status), household factors (wealth, family size, and container used to store water), and school factors (participation in school feeding program). Covariate variables (e.g., sex, age, and wealth) with P values >.25 in the bivariate regression model were retained in the final model to control for confounding.

Variables in model V for *A*. *lumbricoides* infection included individual child factors (sex, age, nail trimming, dirt in fingernail, loss of appetite in past month, handwashing with soap after using latrine, de-worming treatment in past 6 months, and anemia), individual parent factors (mother’s educational status), household factors (wealth and source of drinking water), and school factors (participation in school feeding program). Covariate variables (e.g., sex, nail trimming, and wealth) with P values >.25 in the bivariate regression model were retained in the final model to control for confounding.

### Zero-inflated negative binomial regression for modelling infection intensity

To examine potential factors associated with infection intensity, a count model was applied using the fecal egg counts for *T*. *trichiura* and *A*. *lumbricoides* infections. A Poisson model was appropriate for count data, but the assumption of equal variance and mean did not fit to our data, because the mean of *A*. *lumbricoides* and *T*. *trichiura* eggs was higher than the variance. We have evidence of over-dispersion for *T*. *trichiura A*. *lumbricoides* egg counts. Moreover, zero fecal egg counts for these two infections were more than expected if a Poisson distribution was used [45]. Furthermore, excess number of zeros in our data exceeded those expected under a standard negative-binomial (NB) distribution [45]. Thus, one-part models tend to underestimate the frequencies of zeros and to bias estimation of the covariate effect size [46]. The Vuong test favors ZINB over NB for *T*. *trichiura* (Z = 17.5; P < .001), and for *A*. *lumbricoides* (Z = 9.4; P < .001) eggs count model, indicating the presence of excess zeroes to be accounted. We thus choose ZINB as the best fitting model. The ZINB model is a two-part model that models an over-dispersed count outcomes with inflated zeros [47, 48]. The ZINB model assumes that the excess zero counts come from a logit model and the counts from a negative binomial model [49]. In other words, the excess zeros in *T*. *trichiura* and *A*. *lumbricoides* egg count among participants were generated from two separate process. The first process produced only zero counts, corresponds to participants that are free of *T*. *trichiura* and *A*. *lumbricoides* egg, zero counts for these group of participants considered as true zeros. The second process consisted of participants where *T*. *trichiura* and *A*. *lumbricoides* egg is actually present but not reported due to sampling zeros or diagnostic technique, zero counts for these group of participants considered as false zeros.

### Measure of effect and model fitness

Results were calculated as crude odds ratios and adjusted odds ratios with a 95% CI. Predictors with P values < .05 in the final multilevel, multivariate regression model were reported as statistically significant. The Vuong test was used to compare the ZINB model with a standard negative binomial model and a likelihood ratio test to compare ZINB with a zero-inflated Poisson (ZIP) regression model. The Vuong and likelihood ratio tests with P < .05 favored the ZINB model [46]. Model fitness was checked using -2 log likelihood (deviance) and Akaike information criterion (AIC). The model with the lowest deviance and AIC was used as the final model [46, 50].

## Result

The mean age of the 861 schoolchildren (483 boys and 378 girls) was 11.4 (95% CI: 11.3–11.5) years, ranging from 7 to 14 years. The majority (89.2%; 768) of children lived with their biological parents. About 88.4% (761/861) of mothers and 48.8% (420/861) of fathers never attended school. More than half of the mothers (54.0%; 463) were housewives, and most (77.4%; 619) fathers were farmers. Among heads of households, 91.4% (787/861) were men, and 8.6% (74/861) were women. Family size ranged from 3 to 14 (mean 6.7). Among the participants, 33.3% (287) lived in poor households (S1 Table). About 32% (278/861, 95% CI: 29.2–35.4) of children were stunted, and 9.9% (85, 95% CI: 7.9, 11.9] were thin. Anemia was also occurred in 29.6% (240/810, 95% CI: 26.5–32.8) of children; 85% (204) had mild anemia, 15% (36) had moderate anemia, but none had severe anemia (S2 Table).

As shown in Table 1, more than half (470/850) of any helminths cases were detected using Kato-Katz method, and 44.9% (382/850) of cases were detected using FEC method. Overall, helminths infection were detected in 56.4% (479/850) of children using these two methods. The most frequent helminths were *T*. *trichiura* (42.4%; 360/850), followed by *A*. *lumbricoides* (18.7%; 159/850), *Taenia* species (10.2%; 87/850), hookworm species (4.4%; 37/850), *S*. *stercoralis* (2.5%; 21/850), and *H*. *nana* (0.2%; 2/850). The mean egg intensity was 156.4 epg for *T*. *trichiura* (95% CI: 127.1–185.7), 284.7 epg for *A*. *lumbricoides* (95% CI: 119.5–449.9), 134.4 epg for *Taenia* species (95% CI: 117.4–151.4), 83.7 epg for hookworm (95% CI: 68.3–99.1). Almost all diagnosed infections were light intensity. Only two children had moderate intensity of infection for *T*. *trichiura* and one child for A. lumbricoides. Infection with a single helminth infection was more common 39.6% (337/850) than multiple infections 16.7% (142/850). About 12% (101) of children had double infections and 4.4% (37) had triple infections. We observed *T*.*trichiuria* and *A*. *lumbricoides* co-infection in 10.6% (90) of children (S3 Table).

**Table 1:** Helminths species detected by Kato-Katz and FEC method among schoolchildren in the Wonago district, Southern Ethiopia, 2017 (n = 850).

Helminths species	Kato-Katz	Kato-Katz	FEC	Either Kato-Katz or FEC
	Positive	Positive	Positive	Positive
	n (%)	Mean (SD) of EPG	n (%)	n (%)
*T*.*trichiuria*	342 (40.2)	156.4 (275.3)	278 (32.7)	360 (42.4)
*A*.*lumbricoides*	145 (17.1)	284.7 (1006.3)	130 (15.3)	159 (18.7)
*Taenia* species	85 (10.0)	134.4 (78.9)	44 (5.2)	87 (10.2)
Hookworm species	37 (4.4)	83.7 (46.2)	11 (1.3)	37 (4.4)
*S*.*stercoralis*	12 (1.4)		21 (2.5)	21 (2.5)
*H*.*nana*	0		2 (0.2)	2 (0.2)
Any helminths	470 (55.3)		382 (44.9)	479 (56.4)

EPG: Egg per gram of stool; FEC: Formalin-ether concentration

Any helminths: *T*. *trichiura*, *A*. *lumbricoides*, *Taenia* species, hookworm species, *S*. *stercoralis*, *H*. *nana*

As indicated in Table 2, the proportion of any helminths infection were 57.6% (276/479) among boys, and 55.8% (387) were among children aged 10–14 years. Anemia occurred among 63% (150) of children infected with any helminths. More than two-thirds (68.7%) of thin children were infected with any helminths. The S4 Table of supplementary data shows the proportions of children infected with any helminths, *T*. *trichiura* and *A*. *lumbricoides* in relation to individual, household, and school factors.

**Table 2:** Distribution of helminths infection in relation to age, sex, nutritional statuses and anemia among schoolchildren in the Wonago district, Southern Ethiopia, 2017.

Variables		*T*. *trichiuria*	*A*. *lumbricoides*	*Taenia* species	Hookworm species	Any helminths
Individual child factors	N	n (%)	n (%)	n (%)	n (%)	n (%)
Boys	479	206 (43.0)	96 (20.0)	57 (11.9)	26 (5.4)	276 (57.6)
Girls	371	154 (41.5)	63 (17.0)	30 (8.1)	11 (3.0)	203 (54.7)
7–9	157	72 (45.9)	22 (14.0)	11 (7.0)	12 (7.6)	92 (58.6)
10–14	693	296 (41.6)	137 (19.8)	76 (11.0)	25 (3.6)	387 (55.8)
Not stunted	578	241 (41.7)	110 (19.0)	65 (11.3)	25 (4.3)	326 (56.4)
Stunted	272	119 (43.7)	49 (18.0)	22 (8.1)	12 (4.4)	153 (56.3)
Not thin	767	314 (40.9)	141 (18.4)	79 (10.3)	34 (4.4)	422 (55.0)
Thin	83	46 (55.4)	18 (21.7)	8 (9.6)	3 (3.6)	57 (68.7)
Non anemic	567	225 (39.7)	92 (16.2)	55 (9.7)	20 (3.5)	306 (54.0)
Anemic	238	115 (48.3)	64 (26.9)	30 (12.6)	17 (7.1)	150 (63.0)
Total		360 (42.4)	159 (18.7)	87 (10.2)	37 (4.4)	479 (56.4)

Any helminths: *T*. *trichiura*, *A*. *lumbricoides*, *Taenia* species, hookworm species, *S*. *stercoralis*, *H*. *nana;*

N: children examined; n: children positive with helminths infection

A large proportion of children 81.3% (691/850) for *A*. *lumbricoides*, and 57.6% (490/850) for *T*. *trichiura* were “zero egg excretors.” The median and interquartile range (IQR) of eggs per gram (epg) of stool was 120 (72–168) for *T*. *trichiura*. According to gender, either boys or girls 120 (72–168) had equal median (IQR) of epg for *T*. *trichiura*. Among the age groups, the median (IQR) of epg for *T*. *trichiura* was 120 (72–192) in children aged 7–9 years. The median (IQR) of epg was 120 (72–216) for *A*. *lumbricoides*. The median (IQR) of epg for *A*. *lumbricoides* was 120 (96–216) among boys, and 120 (72–144) in children aged 7–9 years. The S5 and S6 Tables summarize the mean, median, standard deviation, and interquartile range of *T*. *trichiura* and *A*. *lumbricoides* infections for each exposure variable. The S1 Dataset of supplementary data shows the raw data for continuous variables.

### Inter-rater agreement for helminths data

We performed reliability test using 10% of 850 sample. The inter-rater agreement of the two readers either Kato-Katz or FEC was checked with Kappa statistics. In case of discordant results, a third reader was used to confirm the analysis. We observed a good agreement between the initial reading and re-reading in either Kato-Katz or FEC method (P < .001). The Kappa values in the Kato-Katz reading were as follows: *A*. *lumbricoides*, 0.83 [95% CI: 0.72–0.95]; *T*. *trichiura*, 0.88 [95% CI: 0.78–0.98]; *Taenia species*, 0.87 [95% CI: 0.76–0.98]; and hookworm, 0.86 [95% CI: 0.74–0.98]. Among discordant results between the two readers, 7 were for *A*. *lumbricoides*, 5 for *T*. *trichiura*, 5 for *Taenia*, and 5 for hookworm. The proportion of false positive results in the Kato-Katz smear, 1.2% (1/85) were for *A*. *lumbricoides*, 5.9% (5/85*)* for *T*. *trichiura*, 3.5% (3/85) for *Taenia*, and 2.4% (2/85) for hookworm. The proportion of false negative results in the Kato-Katz smear, 7.1% (6/85) were for *A*. *lumbricoides*, 0 for *T*. *trichiura*, 2.4% (2/85) for *Taenia*, 3.5% (3/85) for hookworm. According to the WHO guideline, in the Kato-Katz smear, helminth egg counts between initial reading and second quality control reading was compared. Discrepancies in helminth egg counts in the Kato-Katz smear were detected, 8.2% (7/85) for *A*. *lumbricoides*, 5.9% (5/85) for *T*. *trichiura*, and 5.9% (5/85) for *Taenia* species. For hookworm, the observed differences in egg counts did not exceed 4 eggs per slide (S7 Table).

The Kappa values in the FEC reading were as follows: *A*. *lumbricoides*, 0.91 [95% CI: 0.82–0.99]; *T*. *trichiura*, 0.86 [95% CI: 0.75–0.98]; *Taenia* species, 0.84 [95% CI: 0.71–0.97]; hookworm, 0.86 [95% CI: 0.67–1.00]; and *S*. *stercoralis*, 0.81 [95% CI: 0.62–0.99]. Among discordant results between the two readers, 4 were for *A*. *lumbricoides*, 5 for *T*. *trichiura*, 5 for *Taenia*, 2 for hookworm, and 4 for *S*. *stercoralis*. The proportion of false positive results in the FEC method, 2.4% (2/85) were for *A*. *lumbricoides*, 3.5% (3/85*)* for *T*. *trichiura*, 2.4% (2/85) for *Taenia*, 1.2% (1/85) for hookworm, and 2.4% (2/85) for *S*. *stercoralis*. The proportion of false negative results in the FEC method, 2.4% (2/85) were for *A*. *lumbricoides*, 2.4% (2/85) for *T*. *trichiura*, 3.5% (3/85) for *Taenia*, 1.2% (1/85) for hookworm, and 2.4% (2/85) for *S*. *stercoralis* (S8 Table).

### Variation and risk factors for any helminths infection

Predictors of any helminths infection were estimated using a multivariate, multi-level, mixed-effect, logistic regression analysis. The intra-cluster correlation coefficient (ICC) value, calculated in the empty model with no covariate, was 1.2% at the school and class levels and 0.1% in the final model, indicating unexplained variations of any helminths infection prevalence at the school- and class-levels. See the S10 Table of supplementary data for the results of subsequent multilevel models.

In the bivariate, multi-level, mixed-effect, logistic regression model; the following factors had significant associations with any helminths infection: loss of appetite in the past month, thinness, anemia, having a mother or guardian with no formal education, and using an open container for water storage (S9 Table). In the multivariate, multi-level, mixed-effect, logistic regression model analysis, the risk of any helminths infection was higher among children with loss of appetite in the past month (AOR: 1.89 [95% CI: 1.16, 3.08]), thinness (AOR: 1.73 [95% CI: (1.04, 2.90]), anemia (AOR: 1.45 [95% CI: 1.04, 2.03]), a mother or guardian with no formal education (AOR: 2.08 [95% CI: 1.25, 3.47]), and open containers for water storage (AOR: 2.06 [95% CI: 1.07, 3.99]). However, no significant differences were observed between any helminths infection and sex, age, nail trimming, handwashing before meals, eating undercooked vegetables, wealth, source of drinking water, using treated water at home, or participation in a school feeding program. Table 3 and S10 Table shows the details.

**Table 3:** Multivariate, multilevel, mixed-effect, logistic regression analysis of any helminths infection among schoolchildren in the Wonago district of Southern Ethiopia, 2017.

Variables		Any helminths	Adjusted OR (95% CI)				
Individual child factors		Yes (%)	Model I	Model II	Model III	Model IV	Model V
Sex	Boys	276 (57.6)	**-**	1.0	1.0	1.0	1.0
	Girls	203 (54.7)	**-**	0.96 (0.72, 1.28)	0.97 (0.72, 1.30)	0.98 (0.74, 1.32)	0.98 (0.74, 1.32)
Age in years	7–9	92 (58.6)	**-**	1.19 (0.82, 1.74)	1.17 (0.81, 1.71)	1.13 (0.77, 1.65)	1.13 (0.77, 1.65)
	10–14	387 (55.8)	**-**	1.0	1.0	1.0	1.0
Loss of appetite in past month	Yes	82 (67.8)	**-**	1.77 (1.10, 2.85)*	1.89 (1.18, 3.04)*	1.89 (1.16, 3.08)*	1.89 (1.16, 3.08)*
	No	397 (54.5)	**-**	1.0	1.0	1.0	1.0
Thinness	No	422 (55.0)	**-**	1.0	1.0	1.0	1.0
	Yes	57 (68.7)	**-**	1.73 (1.04, 2.88)*	1.68 (1.01, 2.79)*	1.73 (1.04, 2.90)*	1.73 (1.04, 2.90)*
Anemia	No	306 (54.0)	**-**	1.0	1.0	1.0	1.0
	Yes	150 (63.0)	**-**	1.52 (1.09, 2.12)*	1.49 (1.07, 2.07)*	1.45 (1.04, 2.03)*	1.45 (1.04, 2.03)*
**Individual parent factors**							
Mother’s education level	Never entered school	392 (58.5)	-	-	2.07 (1.26, 3.41)**	2.08 (1.25, 3.47)**	2.08 (1.25, 3.47)**
	Read and write only	47 (58.0)	-	-	1.97 (0.97, 4.02)	1.91 (0.92, 3.97)	1.91 (0.92, 3.97)
	Primary and above	38 (40.0)	-	-	1.0	1.0	1.0
**Household factors**							
Wealth	Poor	165 (57.9)	-	-	-	0.99 (0.69, 1.43)	0.99 (0.69, 1.43)
	Middle	165 (56.3)	-	-	-	1.08 (0.74, 1.58)	1.08 (0.74, 1.58)
	Rich	149 (54.8)	-	-	-	1.0	1.0
Water storage container	Closed container	440 (55.3)	-	-	-	1.0	1.0
	Open container	39 (72.2)	-	-	-	2.06 (1.07, 3.99)*	2.06 (1.07, 3.99)*
**School factor**							
Participates in school food program	No	250 (58.7)	-	-	-	-	1.0
	Yes	229 (54.0)	-	-	-	-	0.98 (0.66, 1.46)
**Model fitness**							
-2log likelihood			1160	1076	1062	1057	1056
AIC			1165	1104	1089	1091	1093

AIC: Akaike information criterion; CI: confidence interval; OR: odds ratio

**P < .01

*P < .05

### Variation and risk factors for *T*. *trichiura* and *A*. *lumbricoides* infections

The intra-cluster correlation value calculated in Model V for *T*. *trichiura* was low and insignificant, indicated that the variability in this infection prevalence was not attributable to class or school factors. The S11 Table of supplementary data shows the results of these tests. Similarly, the variability in *A*. *lumbricoides* prevalence at the school-level was insignificant. However, the intra-cluster correlation value calculated in Model V for *A*. *lumbricoides* infection indicated that 3.2% of the variability in this infection prevalence was attributable to class factors. The S12 Table of supplementary data shows the results of these tests.

For *T*. *trichiura* model, all significant variables in the bivariate, multilevel, mixed-effect model also were significant in the multivariate model. The risk of *T*. *trichiura* infection was higher among children with loss of appetite in the past month (AOR: 1.76 [95% CI: 1.15, 2.71]), thinness (AOR: 1.73 [95% CI: 1.07, 2.78]), anemia (AOR: 1.53 [95% CI: 1.11, 2.12]), a mother or guardian with no formal education (AOR: 1.94 [95% CI: 1.18, 3.19]), and participation in the school food program (AOR: 1.55 [95% CI: 1.13, 2.12]). Furthermore, there were no statistically significant differences between *T*. *trichiura* infection and sex, age, nail trimming, handwashing, eating uncooked vegetable, receiving de-worming treatment in the past 6 months, or wealth (S9 and S11 Tables).

For *A*. *lumbricoides* model, all significant variables in the bivariate, multilevel, mixed-effect model also were significant in the multivariate model. The odds of *A*. *lumbricoides* infection increased by 92% among anemic children (AOR: 1.92 [95% CI: 1.29, 2.88]). The odds were lower among children who received de-worming treatment in the past 6 months [AOR: 0.57 (95% CI: 0.33, 0.98)] and who used water from a protected source [AOR: 0.46 (95% CI: 0.28, 0.77)]. There were no statistically significant differences between *A*. *lumbricoides* infections and age, nail trimming, handwashing, wealth, source of drinking water, and participation in a school food program. The S9 and S12 Tables of supplementary data shows the results.

### Zero-inflated negative binomial regression

#### Model fitness

The alpha dispersion parameter was significant for *T*. *trichiura* at 0.53 (95% CI: 0.45–0.61) and for *A*. *lumbricoides* infection at 0.47 (95% CI: 0.05, 0.37), indicating strong over-dispersion. For *T*. *trichiura* fecal egg count data; comparing the zero-inflated Poisson (ZIP) with a ZINB model, we observed a significant likelihood ratio test at (alpha = 0; Chi square = 38000; P < .001), indicating a ZINB model is best fitted for this data. Meanwhile, the Vuong test favors ZINB over a standard negative binomial (NB) for *T*. *trichiura* (Z = 17.5; P < .001) eggs count model, indicating the presence of excess zeroes to be accounted. The AIC and deviance results for *T*. *trichiura* show that the ZINB model offer a better fit compared to ZIP model (AIC = 4902 for ZINB and 43,186 for ZIP; deviance = 43,126 for ZIP and 4840 for ZINB) (S12 Table). For *A*. *lumbricoides* fecal egg count data; comparing the ZIP with a ZINB model, we observed a significant likelihood ratio test at (alpha = 0; Chi square = 21000; P < .001), indicating a ZINB model is best fitted. The Vuong test also favors ZINB over NB for *A*. *lumbricoides* (Z = 9.4; P < .001) eggs count model, indicating the presence of excess zeroes to be accounted. The AIC and deviance results for *A*. *lumbricoides* also show that the ZINB model offer a better fit compared to ZIP model (AIC = 2494 for ZINB and 23,845 for ZIP; deviance = 23,788 for ZIP and 2436 for ZINB) (S13 Table).

#### Negative binomial count model for *T*. *trichiura* and *A*. *lumbricoides* infections

Intensity of *T*. *trichiura* infection increased among girls (AOR: 1.23 [95% CI: 1.04, 1.45]), and in those using open container for water storage at home (AOR: 1.59 [95% CI: 1.14, 2.22]). The intensity of infection with *A*. *lumbricoides* (AOR: 1.08 [95% CI: 1.01, 1.16]) increased with increasing age. Unclean fingernails (AOR: 1.47 [95% CI: 1.07, 2.03]) were associated with increased intensity of *A*. *lumbricoides* infection. A habit of nail trimming (AOR: 0.56 [95% CI: 0.39, 0.79]) and handwashing with soap after using the latrine (AOR: 0.68 [95% CI: 0.48, 0.95]) lowered the intensity of *A*. *lumbricoides*. The intensity of *A*. *lumbricoides* was higher among children in school feeding programs (AOR: 1.97 [95% CI: 1.49, 2.61]) (Tables 4 and 5).

**Table 4:** Zero-inflated negative binomial regression model for *T*. *trichiura* fecal egg count among schoolchildren in the Wonago district, Southern Ethiopia, 2017 (n = 850).

Variables	Zero-inflated negative binomial model		
	Negative binomial part	Zero-inflated part	
Individual child, parent, household and school factors	Infection intensity AOR (95% CI)	Infection probability AOR (95% CI)	
Sex	Boys	1.0	1.0
	Girls	1.23 (1.04, 1.45)*	1.06 (0.79, 1.43)
Age in years	Mean (SD)	1.01 (0.97, 1.05)	1.07 (0.98, 1.15)
Fingernails trimmed	Yes	0.82 (0.61, 1.09)	0.92 (0.57, 1.49)
	No	1.0	1.0
Habit of eating uncooked vegetable	Yes	1.05 (0.86, 1.27)	0.70 (0.50, 0.99)*
	No	1.0	1.0
Loss of appetite in past month	Yes	0.95 (0.75, 1.21)	0.52 (0.34, 0.81)**
	No	1.0	1.0
Hemoglobin concentration	Mean (SD)	0.96 (0.91, 1.01)	1.13 (1.03, 1.25)*
Thinness	No	1.0	1.0
	Yes	1.10 (0.86, 1.41)	0.59 (0.36, 0.94)*
Mother’s education level	Never entered school	1.18 (0.86, 1.61)	0.56 (0.34, 0.92)*
	Read and write only	1.19 (0.80, 1.78)	0.64 (0.32, 1.26)
	Primary and above	1.0	1.0
Wealth status	Poor	0.99 (0.81, 1.22)	0.98 (0.68, 1.41)
	Middle-class	0.92 (0.74, 1.14)	0.95 (0.66, 1.38)
	Rich	1.0	1.0
Water storage	Closed container	1.0	1.0
	Open container	1.59 (1.14, 2.22)**	0.82 (0.44, 1.49)
Participates in school food program	No	1.0	1.0
	Yes	1.18 (0.98, 1.42)	0.56 (0.41, 0.78)**

AOR: adjusted odds ratio; CI: confidence interval

**P < .01

*P < .05

**Table 5:** Zero-inflated negative binomial regression model for *A*. *lumbricoides* fecal egg count among schoolchildren in the Wonago district, Southern Ethiopia, 2017 (n = 850).

Variables		Zero-inflated negative binomial model	
		Negative binomial part	Zero-inflated part
Individual child, parent, household and school factors		Infection intensity AOR (95% CI)	Infection probability AOR (95% CI)
Sex	Boys	1.03 (0.81, 1.32)	0.96 (0.66, 1.41)
	Girls	1.0	1.0
Age in years	Mean (SD)	1.08 (1.01, 1.16)*	0.90 (0.81, 0.99)*
Fingernails trimmed	Yes	0.56 (0.39, 0.79)**	0.66 (0.36, 1.20)
	No	1.0	1.0
Unclean fingernails	Yes	1.47 (1.07, 2.03)*	0.64 (0.37, 1.08)
	No	1.0	1.0
Handwashing with soap after latrine use	Always	0.94 (0.61, 1.44)	0.68 (0.35, 1.30)
	Sometimes	0.68 (0.48, 0.95)*	1.39 (0.85, 2.27)
	Never	1.0	1.0
De-worming drug in past 6 months	Yes	1.03 (0.76, 1.41)	1.68 (1.01, 2.78)*
	No	1.0	1.0
Hemoglobin concentration	Mean (SD)	0.99 (0.91, 1.07)	1.20 (1.06, 1.36)**
Mother’s education level	Never entered school	1.10 (0.66, 1.85)	0.45 (0.21, 0.96)*
	Read and write only	0.89 (0.49, 1.64)	0.33 (0.13, 0.84)*
	Primary and above	1.0	1.0
Wealth status	Poor	1.17 (0.87, 1.58)	1.17 (0.74, 1.86)
	Middle-class	1.17 (0.87, 1.58)	1.22 (0.77, 1.95)
	Rich	1.0	1.0
Participates in school food program	No	1.0	1.0
	Yes	1.97 (1.49, 2.61)***	1.37 (0.87, 2.15)

AOR: adjusted odds ratio; CI: confidence interval

***P < .001

**P < .01

*P < .05

#### Logit model for predicting excess zeros for *T*. *trichiura* and *A*. *lumbricoides* infections

As shown in Tables 4 and 5, the odds of zero epg counts for *T*. *trichiura* (AOR: 1.13 [95% CI: 1.03, 1.25]) and *A*. *lumbricoides* (AOR: 1.20 [95% CI: 1.06, 1.36]) increased with increasing hemoglobin concentrations. The odds of zero epg counts for *T*. *trichiura* decreased for children who ate uncooked vegetables (AOR: 0.70 [95% CI: 0.50, 0.99]), children who reported loss of appetite in the past month (AOR: 0.52 [95% CI: 0.34, 0.81]), thin children (AOR: 0.59 [95% CI: 0.36, 0.94]), a mother or guardian with no formal education (AOR: 0.56 [95% CI: 0.34, 0.92]), and children in school feeding programs (AOR: 0.56 [95% CI: 0.41, 0.78]). Meanwhile, the odds of zero epg counts for *A*. *lumbricoides* eggs decreased with increasing age (AOR: 0.90 [95% CI: 0.81, 0.99]), whereas the odds increased among children who had received a de-worming drug in the past 6 months (AOR: 1.68 [95% CI: 1.01, 2.78]). However, no significant difference was observed between *T*. *trichiura* infections and age, nail trimming, or wealth. No statistically significant differences were observed between *A*. *lumbricoides* infections and sex, mother’s educational, or wealth.

## Discussion

Helminths infection were found to be a public health problem among schoolchildren aged 7 to 14 years in the Gedeo zone of Southern Ethiopia. Controlling for clustering effects at the school and class levels and accounting for excess zeros of fecal egg counts, we found an association between helminths infection and the following variables: age, thinness, anemia, loss of appetite in past month, unclean fingernails, lack of nail trimming, lack of hand washing with soap after using the latrine, de-worming treatment, mothers’ education levels, water source, and using uncovered water storage container at home. Variations attributable to both class and school-level factors for helminth infections prevalence were less than 5%, indicating minor influence.

We used a large and representative sample of schoolchildren and applied a multilevel, mixed-effect model and a ZINB model to identify risk factors for prevalence and intensity of helminths infection. We examined nutritional status, and measured hemoglobin concentrations. To enhance the detection rate of helminth infections, we used two standard techniques, the Kato-Katz and FEC. Helminths detection and species identification require qualified laboratory expert, and thus we also reported the agreement level of the readers in either method. The dependence of clustered data within the school and class levels was measured and indicated using intra-cluster correlation coefficient. Unlike previous studies [8, 11–17], we reported the intensity of helminth infections and modelled the intensity of helminths egg using a ZINB count model. We determined the fit using deviance and AIC for each model.

Because of the cross-sectional nature of this study, causality between the outcome and the exposure variable cannot be determined with certainty. Multiple stool samples from each child could have enhanced the detection rate of helminths infection [51]. Unfortunately, we did not take multiple samples, due to logistics constraints, but we used two different techniques to analyze a single stool sample, which could have enhanced the detection rate. There is no a ‘gold standard’ test (with 100% accuracy) for diagnosing helminth infections, but it is recommended to use a combined test to improve the detection rate of helminth infections [52, 53]. The Kato-Katz and FEC techniques are recognized for soil-transmitted helminths detection [54]. Moreover, the Kato-Katz method is suitable for quantification of the helminths eggs. However, the duration of the examination and the number of Kato-Katz smears are known to have impact on the sensitivity of Kato-Katz method for hookworm detection [52]. Thus, the low sensitivity of the Kato-Katz technique for hookworm detection, may have underestimated the prevalence of hookworm infection in this study. The performance of the Kato-Katz and FEC method for helminths detection varied in several studies. In this study, the performance of the Kato-Katz method slightly better than the FEC method, and in agreement with previous studies in Ethiopia [55, 56] and a review done by Nikolay et al. [57]. In contrast, Speich et al. and Glinz et al. [58, 59] showed similar or slightly higher sensitivity of the FEC method compared to the Kato-Katz method for helminth infections. The influence of the ether-based concentration techniques on helminth egg count require further studies [59]. Accurate counting of helminth eggs is challenging, it is not uncommon to detect discordant results in helminths diagnosis (e.g. helminth eggs can confuse with eggs from other helminths species or recording errors on the entry forms) [37, 52]. There was good agreement between the initial reading and re-reading from 10% of test result in either Kato-Katz or FEC method. In agreement with Speich et al. [37] the detected false positive results was higher for *T*. *trichiura* than for *A*. *lumbricoides* and the false negative results was higher for *A*. *lumbricoides* than for *T*. *trichiura* in the Kato-Katz method. A combined diagnostic methods, the Koga-agar-plate culture and Baermann are suggested methods for detection of *S*. *stercoralis* [51]. Neither Kato-Katz nor FEC methods are recommended for *S*. *stercoralis* diagnosis. Thus, we have probably underestimated the prevalence of *S*. *stercoralis*. Furthermore, recall bias could be introduced, while using a questionnaire to gather information on the past de-worming history. Moreover, due to homogeneity issues, we were unable to model most school-level risk factors in this study (e.g., sanitation and hygiene facilities were similar across all schools).

The prevalence of any helminths infection that we found was 56.4% align with the findings of 60% and 63% in Southern Ethiopia [17, 60]. However, it is higher than the 23% [24], and 27.7% [12] reported in another studies in Southern Ethiopia. Compared with the 24.6% prevalence found in Ethiopia [16], 26.3% in the Democratic Republic of Congo, 26.5% found in Kenya [61, 62], 10.7% found in Burkina Faso [63], and 10% of global report [64], we found a higher prevalence of 42.4% *T*. *trichiura* infection. The rate of *A*. *lumbricoides* infection 18.7% align with the global report of 20% [64]. However, the rates that we found are higher compared with rates of 10.6% to 13% in other areas of Southern Ethiopia [12, 65]. The rate of 4.4% for hookworm infection in this study aligned with the 7.4% rate found during national mapping [65]. However, it is lower than the 18% in Southern Ethiopia [24], 46.9% [66], and 56.8% [16] reported in other regions of Ethiopia, and 18% of global report [64]. All detected infections in this study were of light intensity [44], which is comparable with other studies in Ethiopia [24, 67, 68]. The rate of multiple helminth infections in this study was 16.7%, and higher than compared to what was reported in the Butajira town 2.6% [69] and in the Bahir Dar 6.3% [70]. Such variations in helminths prevalence could be due to different diagnostic techniques. For instance, among studies reporting low prevalence of any helminths infection in the same region of our study; some used only a single Kato-Katz method [24] and other used a wet mount (low sensitive) technique [12, 71]. In addition, the variations in helminths distribution might be due to difference in the studies’ ecological settings such as altitude, soil type, rainfall, and land surface temperature [72].

We found a moderately high prevalence of 32% for stunting comparable with the values of 28% in the Southern Ethiopia [24], 30.7% in the Filtu Town [73], 32.9% in the Fogera district [30]. The rate of thinness that we found (9.9%) was also similar with the rates (11.6% to 14.0%) found by other studies in Southern Ethiopia [24, 60, 74] but are lower (19.6% to 27.6%) than studies documented in Northern Ethiopia [70, 75, 76]. Despite a well-documented link between soil-transmitted helminth infections and under-nutrition [64, 77], the evidence regarding this association varies. Some studies have reported the same risk of thinness and stunting among infected and non-infected children [11, 76, 78]. In agreement with other studies [70, 75], our study revealed higher rates of any helminth infections among thin children. These children often lose micronutrients, which can impair nutritional status and growth [77].

The rates of anemia was 29.6% among schoolchildren in this setting; higher than the 22% and 23% rates found in Southern Ethiopia [24, 60]. However, it is lower than the 43% rate found by studies of Southwest Ethiopia [16]. The observed association between helminth infections and anemia was expected, as helminth infections are risk factors for anemia [79–81]. Reduced food intake because of inflammatory reactions induced by lesions in the intestinal mucosa and impaired iron absorption due to worm infections could partly explain this association [69, 77]. Furthermore, we found high rates of helminth infections among children who reported a loss of appetite in the past month. Helminth infections increased among children whose mothers had no formal education, similar to previous studies in Ethiopia [8, 12] and rural Mexico [82]. This could be due to lack of knowledge about poor home sanitation and hygiene.

Using piped water has been shown to influence the prevalence of *A*. *lumbricoides* infection [9]. In this study, low rates of *A*. *lumbricoides* infection were observed among children living in households using a protected water source, indicating a possibility for contamination when water is not protected from soil-transmitted helminths eggs during transport and storage [83].

Our ZINB model indicated that eating uncooked vegetables lowered the probability of remaining free from *T*. *trichiura* infections. Ingesting contaminated raw vegetables could play an important role in transmitting helminths infection [84]. The zero-inflation model also indicated higher probability of children remaining free from *T*. *trichiura* and *A*. *lumbricoides* infections as their hemoglobin concentrations increase. The observed infection intensity in this study was light, and although light infection by *T*. *trichiura* and *A*. *lumbricoides* may not be enough to produce significant blood loss, it may aggravate the condition [85]. However, de Gier et al. found low hemoglobin concentrations among children with light *T*. *trichiura* infections [80]. This finding could be affected by unmeasured factors, such as low dietary iron intake and malaria. In the zero-inflation model, we also observed a high risk of helminths infection, particularly *T*. *trichiura* infection, among children in households using open containers for water storage. Similar findings have been observed in Kenya [61]. The high percentage of unimproved water sources and the practice of open defecation, particularly in rural Ethiopia, offer support for this finding [86]. We indeed observed a high percentage of unimproved toilet facilities in the study households.

Using the ZINB model, we observed increased intensity of *A*. *lumbricoides* infections and a decline in the probability of older children remaining free from this infection. Older children may participate in activities and environments that make them more prone to infection than younger children. In contrast, a previous study has reported a lower risk of helminths infection in the older age group [87]. This reduced risk could be due to immunological and behavioral factors related to hygiene [88].

Nail and hand hygiene are well known individual factors affecting helminths infection prevalence and intensity [89]. We found an increased intensity of *A*. *lumbricoides* infection among children with unclean finger nails, as has been reported by others [12, 15]. Nail trimming and handwashing with soap after using the latrine led to reduced intensity of *A*. *lumbricoides* infection, similar to findings in other studies [8, 9, 14, 90]. Eating uncooked vegetables has been reported as a risk factor for helminths infection [87]. Furthermore, receiving de-worming drugs in the past 6 months significantly increased the probability of children remaining free from *A*. *lumbricoides*, as has been observed in rural Bangladesh [91].

Children participating in school feeding programs had high rates of *T*. *trichiura* infection and increased intensity of *A*. *lumbricoides* infection. This finding suggests unsafe or unhygienic food preparation and poor sanitary facilities at schools in the study area. School sanitation and hygiene could affect this finding, though we were unable to show a link due to similarity of this potential exposure variable. Furthermore, some schools had no access to safe water, putting those children at higher risk. Schools with feeding programs thus may be area at high risk of food insecurity and vulnerability to infection.

Although Ethiopia launched a national school-based de-worming program in 2015, soil-transmitted helminth infection remain high among schoolchildren in the rural areas. Variations attributable to both class- and school-level factors for helminths infection prevalence were low. Most individual and few household factors were found to be important predictors for helminths infection prevalence and intensity, and high rates of *T*. *trichiura* infection and intensity of *A*. *lumbricoides* among children in school feeding programs also were observed. Interventions that improve hygiene among schoolchildren can reduce the burden of helminths infection in settings such as Gedeo. Access to safe water at school and at home is a crucial part of infection reduction strategies. Periodic de-worming programs in schools must be strengthened. To that end, school teachers should work with health workers to provide health education about personal hygiene. Integrated intervention activities focusing on the individual, household, and school will reduce the burden of helminths infections.

## Supporting information

S1 Checklist STROBE checklist. (DOCX)

S1 Dataset Dataset for continuous variables. (XLSX)

S1 Table Demographic and socio-economic status of schoolchildren and their parents in the Wonago district, Southern Ethiopia, 2017. (DOCX)

S2 Table Stunting, thinness, and anemia among schoolchildren in the Wonago district, Southern Ethiopia, 2017. (DOCX)

S3 Table Distribution of helminths co-infection among schoolchildren in the Wonago district, Southern Ethiopia, 2017. (DOCX)

S4 Table Distribution of helminths infection among schoolchildren in the Wonago district, Southern Ethiopia, 2017. (DOCX)

S5 Table The mean, median, SD, and IQR of *T*. *trichiuria* infection loads in egg per gram of stool among schoolchildren in the Wonago district, Southern Ethiopia, 2017. (DOCX)

S6 Table The mean, median, SD, and IQR of *A*. *lumbricoides* infection loads in egg per gram of stool among schoolchildren in the Wonago district, Southern Ethiopia, 2017. (DOCX)

S7 Table Inter-rater agreement for the readings of 85 Kato-Katz microscopic slides. (DOCX)

S8 Table Inter-rater agreement for the readings of 85 FEC microscopic slides. (DOCX)

S9 Table Bivariate, multilevel, mixed-effect, regression analysis of any helminths, *T*. *trichiuria*, and *A*. *lumbricoides* among schoolchildren in the Wonago district, Southern Ethiopia, 2017. (DOCX)

S10 Table Multivariate, multilevel, mixed-effect, regression analysis of any helminths infection among schoolchildren in the Wonago district, Southern Ethiopia, 2017. (DOCX)

S11 Table Multivariate, multilevel, mixed-effect, logistic regression analysis of *T*. *trichiuria* infection among schoolchildren in the Wonago district, Southern Ethiopia, 2017. (DOCX)

S12 Table Multivariate, multilevel, mixed-effect, logistic regression analysis of *A*. *lumbricoides* infection among schoolchildren in the Wonago district, Southern Ethiopia, 2017. (DOCX)

S13 Table Model validation for *T*. *trichiuria* and *A*. *lumbricoides* egg count model. (DOCX)

## References

[1] WHO. Soil-transmitted helminth infections [Internet]. 2019. Available from: https://www.who.int/news-room/fact-sheets/detail/soil-transmitted-helminth-infections.

[2] WHO Ethiopia. Ethiopian school-based deworming campaign targets 17 million children [Internet]. 2015. Available from: https://who.insomnation.com/news/ethiopian-school-based-deworming-campaign-targets-17-million-children.

[3] Johnston EA, Teague J, Graham JP. Challenges and opportunities associated with neglected tropical disease and water, sanitation and hygiene intersectoral integration programs. BMC Public Health. 2015;15: 547 10.1186/s12889-015-1838-7 26062691 PMC4464235

[4] Pullan R, Smith J, Jasrasaria R, Brooker S. Global numbers of infection and disease burden of soil transmitted helminth infections in 2010. Parasit Vectors. 2014;7: 37 10.1186/1756-3305-7-37 24447578 PMC3905661

[5] World Health Organization. Soil-transmitted helminthiases: eliminating as public health problem soil-transmitted helminthiases in children: progress report 2001–2010 and strategic plan 2011–2020 [Internet]. World Health Organization. 2012. Available from: https://apps.who.int/iris/handle/10665/44804.

[6] Tchuem Tchuenté L-A. Control of soil-transmitted helminths in sub-Saharan Africa: Diagnosis, drug efficacy concerns and challenges. Acta Trop. 2011;120 Suppl 1: S4–S11. 10.1016/j.actatropica.2010.07.001 20654570

[7] Kassebaum NJ. The Global burden of anemia. Hematol Oncol Clin North Am. 2016;30(2): 247–308. 10.1016/j.hoc.2015.11.002 27040955

[8] Asemahagn MA. Parasitic infection and associated factors among the primary schoolchildren in Motta Town, Western Amhara, Ethiopia. Am J Public Health Res. 2014;2(6): 248–54.

[9] Strunz EC, Addiss DG, Stocks ME, Ogden S, Utzinger J, Freeman MC. Water, sanitation, hygiene, and soil-transmitted helminth infection: a systematic review and meta-analysis. PLoS Med. 2014;11(3): e1001620–e. 10.1371/journal.pmed.1001620 24667810 PMC3965411

[10] hiwot Y G., Degarege A, Erko B. Prevalence of intestinal parasitic infections among children under five years of age with emphasis on Schistosoma mansoni in Wonji Shoa Sugar Estate, Ethiopia. PLoS One. 2014;9(10): e109793 10.1371/journal.pone.0109793 25296337 PMC4190315

[11] Amare B, Ali J, Moges B, Yismaw G, Belyhun Y, Gebretsadik S, et al Nutritional status, intestinal parasite infection and allergy among schoolchildren in northwest Ethiopia. BMC Pediatr. 2013; 13:7 10.1186/1471-2431-13-7 23311926 PMC3565883

[12] Haftu D, Deyessa N, Agedew E. Prevalence and determinant factors of intestinal parasites among schoolchildren in Arba Minch town, Southern Ethiopia. Am J Health Research. 2014;2(5): 247–54.

[13] Tulu B, Taye S, Amsalu E. Prevalence and its associated risk factors of intestinal parasitic infections among Yadot primary schoolchildren of South Eastern Ethiopia: a cross-sectional study. BMC Res Notes. 2014;7: 848 10.1186/1756-0500-7-848 25425173 PMC4289289

[14] Gelaw A, Anagaw B, Nigussie B, Silesh B, Yirga A, Alem M, et al Prevalence of intestinal parasitic infections and risk factors among schoolchildren at the University of Gondar Community School, Northwest Ethiopia: a cross-sectional study. BMC Public Health. 2013;13: 304 10.1186/1471-2458-13-304 23560704 PMC3621079

[15] Mahmud MA, Spigt M, Mulugeta Bezabih A, Lopez Pavon I, Dinant GJ, Blanco Velasco R. Risk factors for intestinal parasitosis, anaemia, and malnutrition among schoolchildren in Ethiopia. Pathogens and global health. 2013;107(2): 58–65. 10.1179/2047773213Y.0000000074 23683331 PMC4001479

[16] Desalegn Wolide A, Mossie A, Gedefaw L. Nutritional iron deficiency anemia: magnitude and its predictors among school age children, southwest Ethiopia: a community based cross-sectional study. PLoS One. 2018;13(8): e0202380 10.1371/journal.pone.0202380 30092032 PMC6084990

[17] Abossie A, Seid M. Assessment of the prevalence of intestinal parasitosis and associated risk factors among primary schoolchildren in Chencha town, Southern Ethiopia. BMC Public Health. 2014;14: 166 10.1186/1471-2458-14-166 24528627 PMC3933408

[18] Ministry of Finance and Economic Development Federal Democratic Republic of Ethiopia. Assessing progress towards the Millenium Development Goals: Ethiopia MDGs Report Addis Ababa, Ethiopia 2012.

[19] Government of the Federal Democratic Republic of Ethiopia. National Nutrition Programme. June 2013 –June 2015.

[20] Ethiopia Ministry of Health. Health Sector Transformation Plan 2015/16–2019/20. 2015: 31–157.

[21] Ethiopia Ministry Of Health. National Hygiene and Sanitation Strategic Action Plan for Rural, Per-Urban and Informal Settlements in Ethiopia. Addis Ababa, Ethiopia. 2011–2015.

[22] Ministry of Education and UNICEF Ethiopia. Global Out of School Children Initiative. Study on Situation of Out of School Children (OOSC) in Ethiopia. 2012.

[23] UNICEF’S Child-Friendly Schools: Ethiopia case study. Addis Ababa, Ethiopia. 2010.

[24] Grimes JET, Tadesse G, Gardiner IA, Yard E, Wuletaw Y, Templeton MR, et al Sanitation, hookworm, anemia, stunting, and wasting in primary schoolchildren in southern Ethiopia: Baseline results from a study in 30 schools. PLoS Negl Trop Dis. 2017;11(10): e0005948 10.1371/journal.pntd.0005948 28991894 PMC5633169

[25] Alexander N. Review: analysis of parasite and other skewed counts. Trop Med Int Health. 2012;17(6): 684–93. 10.1111/j.1365-3156.2012.02987.x 22943299 PMC3468795

[26] Standard Treatment Guideline for Health Centers. Drug administration and control authority of Ethiopia contents. Addis Ababa, Ethiopia: The Drug Administration and Control Authority (DACA) of Ethiopia; January 2010; 349.

[27] Sullivan KM, Dean A, Soe MM. OpenEpi: a web-based epidemiologic and statistical calculator for public health. Public Health Rep. 2009;124(3): 471–4. 10.1177/003335490912400320 19445426 PMC2663701

[28] Daniel WW. Biostatistics: a foundation for analysis in the health sciences. New York: John Willey and Sons 1999.

[29] Mesfin F, Berhane Y, Worku A. Anemia among primary schoolchildren in Eastern Ethiopia. PLoS One. 2015;10(4): e0123615 10.1371/journal.pone.0123615 25902055 PMC4406736

[30] Mekonnen H, Tadesse T, Kisi K. Malnutrition and its correlates among rural primary schoolchildren of Fogera District, Northwest Ethiopia. J Nutr Disord Ther. 2013: 2–7.

[31] Ali J, Yifru S, Woldeamanuel Y. Prevalence of tinea capitis and the causative agent among schoolchildren in Gondar, North West Ethiopia. Ethiop Med J. 2009;47(4): 261–9. 20067140

[32] Komba EV, Mgonda YM. The spectrum of dermatological disorders among primary schoolchildren in Dares Salaam. BMC Public Health. 2010;10: 765 10.1186/1471-2458-10-765 21162714 PMC3009652

[33] Montresor A, Crompton DWT, Hall A, Bundy DAP, Savioli L. Guidelines for the evaluation of soil-transmitted helminthiasis and schistosomiasis at community level: A guide for managers of control programmes. Geneva: World Health Organization; 1998.

[34] WHO. Basic laboratory methods in medical parasitology. Geneva: World Health Organization; 1991.

[35] Manser MM, Saez ACS, Chiodini PL. Faecal parasitology: Concentration methodology needs to be better standardised. PLoS Negl Trop Dis. 2016;10(4): e0004579 10.1371/journal.pntd.0004579 27073836 PMC4830587

[36] WHO. Assessing the efficacy of anthelminthic drugs against schistosomiasis and soil-transmitted helminthiases. Geneva: World Health Organization; 2013.

[37] Speich B, Ali SM, Ame SM, Albonico M, Utzinger J, Keiser J. Quality control in the diagnosis of Trichuris trichiura and Ascaris lumbricoides using the Kato-Katz technique: experience from three randomised controlled trials. Parasites and Vectors. 2015;8(1): 82. 25652120 10.1186/s13071-015-0702-z PMC4326492

[38] Vyas S, Kumaranayake L. Constructing socio-economic status indices: how to use principal components analysis. Health Policy Plan. 2006;21(6): 459–68. 10.1093/heapol/czl029 17030551

[39] WHO AnthroPlus for personal computers manual. Software for assessing growth of the world's children and adolescents. Geneva: WHO; 2009 http://www.who.int/growthref/tools/en/.

[40] de Onis M, Onyango AW, Borghi E, Siyam A, Nishida C, Siekmann J. Development of a WHO growth reference for school-aged children and adolescents. Bull World Health Organ. 2007;85(9): 660–7. 10.2471/blt.07.043497 18026621 PMC2636412

[41] WHO. Haemoglobin concentrations for the diagnosis of anaemia and assessment of severity. Vitamin and Mineral Nutrition Information System. Geneva, World Health Organization, 2011 (WHO/NMH/NHD/MNM/11.1).

[42] Kirkwood BR, Sterne JAC. Essential Medical Statistics. 2 ed Australia: Blackwell Publishing Ltd; 2003.

[43] Leuenberger A, Nassoro T, Said K, Fenner L, Sikalengo G, Letang E, et al Assessing stool quantities generated by three specific Kato-Katz thick smear templates employed in different settings. Infectious Diseases of Poverty. 2016;5: 58 10.1186/s40249-016-0150-9 27364623 PMC4929780

[44] WHO. Prevention and control of schistosomiasis and soil-transmitted helminthiasis: Report of a WHO expert committee. Geneva; 2002. WHO Technical Report Series, 912. 12592987

[45] Martin T, Wintle B, Rhodes J, Kuhnert P, Field S, Lowchoy S, et al Zero tolerance ecology: Improving ecological inference by modelling the source of zero observations. Ecol Lett. 2005;8: 1235–46. 10.1111/j.1461-0248.2005.00826.x 21352447

[46] Xu L, Paterson AD, Turpin W, Xu W. Assessment and selection of competing models for zero-inflated microbiome data. PLoS One. 2015;10(7): e0129606 10.1371/journal.pone.0129606 26148172 PMC4493133

[47] Forrer A, Vounatsou P, Sayasone S, Vonghachack Y, Bouakhasith D, Utzinger J, et al Risk profiling of hookworm infection and intensity in Southern Lao People’s Democratic Republic using bayesian models. PLoS Negl Trop Dis. 2015;9(3): e0003486 10.1371/journal.pntd.0003486 25822794 PMC4378892

[48] Chipeta MG, Ngwira BM, Simoonga C, Kazembe LN. Zero adjusted models with applications to analysing helminths count data. BMC Res Notes. 2014;7(1): 856 10.1186/1756-0500-7-856 25430726 PMC4289350

[49] Wang C, Torgerson PR, Höglund J, Furrer R. Zero-inflated hierarchical models for faecal egg counts to assess anthelmintic efficacy. Vet Parasitol. 2017;235:20–8. 10.1016/j.vetpar.2016.12.007 28215863

[50] Twisk JWR. Applied multilevel analysis. A practical guides to biostatistics and epidemiology. Cambridge: Cambridge University Press; 2006.

[51] Knopp S, Mgeni AF, Khamis IS, Steinmann P, Stothard JR, Rollinson D, et al Diagnosis of soil-transmitted helminths in the era of preventive chemotherapy: effect of multiple stool sampling and use of different diagnostic techniques. PLoS Negl Trop Dis. 2008;2 10.1371/journal.pntd.0000331 18982057 PMC2570799

[52] Tarafder MR, Carabin H, Joseph L, Balolong E Jr., Olveda R, McGarvey ST. Estimating the sensitivity and specificity of Kato-Katz stool examination technique for detection of hookworms, Ascaris lumbricoides and Trichuris trichiura infections in humans in the absence of a 'gold standard'. Int J Parasitol. 2010;40(4): 399–404. 10.1016/j.ijpara.2009.09.003 19772859 PMC2829363

[53] Knopp S, Salim N, Schindler T, Karagiannis Voules DA, Rothen J, Lweno O, et al Diagnostic accuracy of Kato-Katz, FLOTAC, Baermann, and PCR methods for the detection of light-intensity hookworm and Strongyloides stercoralis infections in Tanzania. Am J Trop Med Hyg. 2014;90(3): 535–45. 10.4269/ajtmh.13-0268 24445211 PMC3945701

[54] Meurs L, Polderman AM, Vinkeles Melchers NVS, Brienen EAT, Verweij JJ, Groosjohan B, et al Diagnosing polyparasitism in a high-prevalence setting in Beira, Mozambique: Detection of intestinal parasites in fecal samples by microscopy and Real-Time PCR. PLoS Negl Trop Dis. 2017;11(1): e0005310–e. 10.1371/journal.pntd.0005310 28114314 PMC5289637

[55] Endris M, Tekeste Z, Lemma W, Kassu A. Comparison of the Kato-Katz, Wet Mount, and Formol-Ether Concentration Diagnostic Techniques for Intestinal Helminth Infections in Ethiopia. ISRN Parasitology. 2013;2013:180439 10.5402/2013/180439 27335845 PMC4890854

[56] Yimer M, Hailu T, Mulu W, Abera B. Evaluation performance of diagnostic methods of intestinal parasitosis in school age children in Ethiopia. BMC Res Notes. 2015;8(1): 820 10.1186/s13104-015-1822-4 26708493 PMC4691533

[57] Nikolay B, Brooker SJ, Pullan RL. Sensitivity of diagnostic tests for human soil-transmitted helminth infections: a meta-analysis in the absence of a true gold standard. Int J Parasitol. 2014;44(11):765–74. 10.1016/j.ijpara.2014.05.009 24992655 PMC4186778

[58] Speich B, Utzinger J, Marti H, Ame SM, Ali SM, Albonico M, et al Comparison of the Kato-Katz method and ether-concentration technique for the diagnosis of soil-transmitted helminth infections in the framework of a randomised controlled trial. Eur J Clin Microbiol Infect Dis. 2014;33(5): 815–22. 10.1007/s10096-013-2019-1 24272064

[59] Glinz D, Silué KD, Knopp S, Lohourignon LK, Yao KP, Steinmann P, et al Comparing Diagnostic Accuracy of Kato-Katz, Koga Agar Plate, Ether-Concentration, and FLOTAC for Schistosoma mansoni and Soil-Transmitted Helminths. PLoS Negl Trop Dis. 2010;4(7): e754 10.1371/journal.pntd.0000754 20651931 PMC2907416

[60] Shaka MF, Wondimagegne YA. Anemia, a moderate public health concern among adolescents in South Ethiopia. PLoS One. 2018;13(7): e0191467 10.1371/journal.pone.0191467 30016373 PMC6049899

[61] Worrell CM, Wiegand RE, Davis SM, Odero KO, Blackstock A, Cuéllar VM, et al A cross-sectional study of water, sanitation, and hygiene-related risk factors for soil-transmitted helminth infection in urban school, and preschool-aged children in Kibera, Nairobi. PLoS One. 2016;11(3): e0150744–e. 10.1371/journal.pone.0150744 26950552 PMC4780697

[62] Matangila JR, Doua JY, Linsuke S, Madinga J, Inocêncio da Luz R, Van Geertruyden J-P, et al Malaria, schistosomiasis and soil transmitted helminth burden and their correlation with anemia in children attending primary schools in Kinshasa, Democratic Republic of Congo. PLoS One. 2014;9(11): e110789–e. 10.1371/journal.pone.0110789 25372029 PMC4220949

[63] Erismann S, Diagbouga S, Odermatt P, Knoblauch AM, Gerold J, Shrestha A, et al Prevalence of intestinal parasitic infections and associated risk factors among schoolchildren in the Plateau Central and Centre-Ouest regions of Burkina Faso. Parasit Vectors. 2016;9(1): 554 10.1186/s13071-016-1835-4 27756339 PMC5069922

[64] Alum A, Rubino JR, Ijaz MK. The global war against intestinal parasites: should we use a holistic approach? Int J Infect Dis. 2010;14(9): e732–8. 10.1016/j.ijid.2009.11.036 20399129

[65] Grimes JET, Tadesse G, Mekete K, Wuletaw Y, Gebretsadik A, French MD, et al School water, sanitation, and hygiene, soil-transmitted helminths, and schistosomes: National mapping in Ethiopia. PLoS Negl Trop Dis. 2016;10(3): e0004515 10.1371/journal.pntd.0004515 26954688 PMC4783033

[66] Yimam Y, Degarege A, Erko B. Effect of anthelminthic treatment on helminth infection and related anaemia among school-age children in northwestern Ethiopia. BMC Infect Dis. 2016;16(1): 613 10.1186/s12879-016-1956-6 27793110 PMC5084399

[67] Gashaw F, Aemero M, Legesse M, Petros B, Teklehaimanot T, Medhin G, et al Prevalence of intestinal helminth infection among schoolchildren in Maksegnit and Enfranz Towns, northwestern Ethiopia, with emphasis on Schistosoma mansoni infection. Parasit Vectors. 2015;8(1): 567. 26520794 10.1186/s13071-015-1178-6 PMC4628278

[68] Habtamu K, Degarege A, Ye-Ebiyo Y, Erko B. Comparison of the Kato-Katz and FLOTAC techniques for the diagnosis of soil-transmitted helminth infections. Parasitol Int. 2011;60(4): 398–402. 10.1016/j.parint.2011.06.020 21726662

[69] Stephenson LS, Holland CV, Cooper ES. The public health significance of Trichuris trichiura. Parasitol. 2000;121 Suppl: S73–S95. 10.1017/s0031182000006867 11386693

[70] Hailegebriel T. Undernutrition, intestinal parasitic infection and associated risk factors among selected primary schoolchildren in Bahir Dar, Ethiopia. BMC Infect Dis. 2018;18(1): 394 10.1186/s12879-018-3306-3 30103696 PMC6090691

[71] Yimer M, Hailu T, Mulu W, Abera B. Evaluation performance of diagnostic methods of intestinal parasitosis in school age children in Ethiopia. BMC Res Notes. 2015;8:820–. 10.1186/s13104-015-1822-4 26708493 PMC4691533

[72] Schüle SA, Clowes P, Kroidl I, Kowuor DO, Nsojo A, Mangu C, et al Ascaris lumbricoides infection and its relation to environmental factors in the Mbeya region of Tanzania, a cross-sectional, population-based study. PLoS One. 2014;9(3):e92032–e. 10.1371/journal.pone.0092032 24643023 PMC3958400

[73] Gutema B, Adissu W, Asress Y, Gedefaw L. Anemia and associated factors among school-age children in Filtu Town, Somali region, Southeast Ethiopia. BMC hematology. 2014;14(1): 13 10.1186/2052-1839-14-13 25170422 PMC4147173

[74] Getachew T, Argaw A. Intestinal helminth infections and dietary diversity score predict nutritional status of urban schoolchildren from southern Ethiopia. BMC Nutrition. 2017;3(1): 9.

[75] Nguyen NL, Gelaye B, Aboset N, Kumie A, Williams MA, Berhane Y. Intestinal parasitic infection and nutritional status among schoolchildren in Angolela, Ethiopia. J Prev Med Hyg. 2012;53(3): 157–64. 23362622 PMC3587130

[76] Abdi M, Nibret E, Munshea A. Prevalence of intestinal helminthic infections and malnutrition among schoolchildren of the Zegie Peninsula, northwestern Ethiopia. J Infect Public Health. 2017;10(1): 84–92. 10.1016/j.jiph.2016.02.009 27026133

[77] Katona P, Katona-Apte J. The interaction between nutrition and infection. Clin Infect Dis. 2008;46(10): 1582–8. 10.1086/587658 18419494

[78] Degarege A, Hailemeskel E, Erko B. Age-related factors influencing the occurrence of undernutrition in northeastern Ethiopia. BMC Public Health. 2015;15: 108 10.1186/s12889-015-1490-2 25885212 PMC4324415

[79] Degarege A, Animut A, Medhin G, Legesse M, Erko B. The association between multiple intestinal helminth infections and blood group, anaemia and nutritional status in human populations from Dore Bafeno, southern Ethiopia. J Helminthol. 2014;88(2): 152–9. 10.1017/S0022149X12000855 23286203

[80] de Gier B, Nga TT, Winichagoon P, Dijkhuizen MA, Khan NC, van de Bor M, et al Species-specific associations between soil-transmitted helminths and micronutrients in Vietnamese schoolchildren. Am J Trop Med Hyg. 2016;95(1): 77–82. 10.4269/ajtmh.15-0533 27246448 PMC4944714

[81] Suchdev PS, Davis SM, Bartoces M, Ruth LJ, Worrell CM, Kanyi H, et al Soil-transmitted helminth infection and nutritional status among urban slum children in Kenya. Am J Trop Med Hyg. 2014;90(2): 299–305. 10.4269/ajtmh.13-0560 24343884 PMC3919237

[82] Quihui L, Valencia ME, Crompton DWT, Phillips S, Hagan P, Morales G, et al Role of the employment status and education of mothers in the prevalence of intestinal parasitic infections in Mexican rural schoolchildren. BMC Public Health. 2006;6: 225–. 10.1186/1471-2458-6-225 16956417 PMC1584408

[83] Khairy AE, El Sebaie O, Abdel Gawad A, El Attar L. The sanitary condition of rural drinking water in a Nile Delta village. I. Parasitological assessment of 'zir' stored and direct tap water. J Hyg. 1982;88(1): 57–61. 10.1017/s0022172400069898 7057027 PMC2134156

[84] Mulambalah C, Ruto J. Prevalence and infection intensity of geohelminthiases among schoolchildren as an environmental health indicator to guide preventive activities in Nandi County, Kenya. Trop J Med Res. 2016;19(2): 131–7.

[85] Wani SA, Ahmad F, Zargar SA, Dar ZA, Dar PA, Tak H, et al Soil-transmitted helminths in relation to hemoglobin status among schoolchildren of the Kashmir Valley. Parasitol. 2008;94(3): 591–3. 10.1645/GE-1400.1 18605794

[86] WHO, UNICEF. Progress on sanitation and drinking water. 2015 update and MDG assessment. Geneva: World Health Organization https://apps.who.int/iris/handle/10665/177752

[87] Feleke BE. Nutritional status and intestinal parasite in school age children: A comparative cross-sectional study. Int J Pediatr. 2016; 1962128 10.1155/2016/1962128 27656219 PMC5021489

[88] O'Lorcain P, Holland CV. The public health importance of Ascaris lumbricoides. Parasitol. 2000;121 Suppl: S51–S71. 10.1017/s0031182000006442 11386692

[89] Mahmud MA, Spigt M, Bezabih AM, Pavon IL, Dinant G-J, Velasco RB. Efficacy of handwashing with soap and nail clipping on intestinal parasitic infections in school-aged children: A factorial cluster randomized controlled trial. PLoS Med. 2015;12(6): e1001837–e. 10.1371/journal.pmed.1001837 26057703 PMC4461173

[90] Shumbej T, Belay T, Mekonnen Z, Tefera T, Zemene E. Soil-transmitted helminths and associated factors among pre-schoolchildren in Butajira Town, South-central Ethiopia: A community-based cross-sectional study. PLoS One. 2015;10(8): e0136342 10.1371/journal.pone.0136342 26305361 PMC4548951

[91] Benjamin-Chung J, Nazneen A, Halder AK, Haque R, Siddique A, Kopor MSUK, et al The interaction of deworming, improved sanitation, and household flooring with soil-transmitted helminth infection in rural Bangladesh. PLoS Negl Trop Dis. 2015;9(12): e0004256 10.1371/journal.pntd.0004256 26624994 PMC4666415

