# Supplementary material for: Helminth infections among rural schoolchildren in Southern Ethiopia: A cross-sectional multilevel and zero-inflated regression model
Source: PLoS Negl Trop Dis. 2020 Dec 22;14(12):e0008002. doi: 10.1371/journal.pntd.0008002 (PMC7755205; doi:10.1371/journal.pntd.0008002)
Supplement: S2 Table — (DOCX) [file pntd.0008002.s004.docx]

S2 Table. Stunting, thinness, and anemia, among schoolchildren in the Wonago district, Southern Ethiopia, 2017

| **Variables** | **Frequency** | **Percent** |
| --- | --- | --- |
| Not stunted | 583 | 67.7 |
| Stunted | 278 | 32.3 |
| Not thin | 776 | 90.1 |
| Thin | 85 | 9.9 |
| Non anemic | 570 | 70.4 |
| Anemic | 240 | 29.6 |
| Mild anemia | 204 | 85 |
| Moderate anemia | 36 | 15 |
