# Supplementary material for: Helminth infections among rural schoolchildren in Southern Ethiopia: A cross-sectional multilevel and zero-inflated regression model
Source: PLoS Negl Trop Dis. 2020 Dec 22;14(12):e0008002. doi: 10.1371/journal.pntd.0008002 (PMC7755205; doi:10.1371/journal.pntd.0008002)
Supplement: S3 Table — (DOCX) [file pntd.0008002.s005.docx]

S3 Table. Distribution of helminths co-infection among schoolchildren in the Wonago district, Southern Ethiopia, 2017 (n=850)

| **Co-infection** | **Frequency** | **Percent** |
| --- | --- | --- |
| No infection | 371 | 43.6 |
| Single infection | 337 | 39.6 |
| Double infections | 101 | 11.9 |
| Triple infections | 37 | 4.4 |
| Quadruple infections | 4 | 0.5 |
| *T. trichiuria,* and *A. lumbricoides* | 90 | 10.6 |
| *T. trichiuria,* and Hookworm | 15 | 1.8 |
| *A. lumbricoides* and Hookworm | 13 | 1.5 |
