# Supplementary material for: Helminth infections among rural schoolchildren in Southern Ethiopia: A cross-sectional multilevel and zero-inflated regression model
Source: PLoS Negl Trop Dis. 2020 Dec 22;14(12):e0008002. doi: 10.1371/journal.pntd.0008002 (PMC7755205; doi:10.1371/journal.pntd.0008002)
Supplement: S4 Table — (DOCX) [file pntd.0008002.s006.docx]

S4 Table. Distribution of helminths infection among schoolchildren in the Wonago district, Southern Ethiopia, 2017 (n=850)

| **Variables** | | **Any helminths** | ***T. trichiuria*** | ***A. lumbricoides*** |
| --- | --- | --- | --- | --- |
| **Individual child factors** | | **Yes (n (%)** | **Yes (n (%)** | **Yes (n (%)** |
| Sex of child | Boys | 276 (57.6) | 206 (43.0) | 96 (20.0) |
|  | Girls | 203 (54.7) | 154 (41.5) | 63 (17.0) |
| Child age in years | 7-9 | 92 (58.6) | 72 (46.0) | 22 (14.0) |
|  | 10-14 | 387 (55.8) | 288 (41.6) | 137 (19.8) |
| Finger nails trimmed | Yes | 386 (55.5) | 296 (42.6) | 128 (18.4) |
|  | No | 93 (60.0) | 64 (41.3) | 31 (20.0) |
| Dirt on children fingers | Yes | 121 (58.5) | 89 (43.0) | 48 (23.2) |
|  | No | 358 (55.7) | 271 (42.2) | 111 (17.3) |
| Hand washing with soap after latrine | Always | 63 (61.8) | 50 (49.0) | 22 (21.6) |
|  | Sometimes | 264 (54.9) | 200 (41.6) | 78 (16.2) |
|  | Never | 152 (56.9) | 110 (41.2) | 59 (22.1) |
| Hand washing with soap before meal | Yes | 466 (56.2) | 349 (42.1) | 155 (18.7) |
|  | No | 13 (61.9) | 11 (52.4) | 4 (19.1) |
| Eats uncooked vegetables | Yes | 125 (60.0) | 98 (47.1) | 40 (19.2) |
|  | No | 354 (55.1) | 262 (40.8) | 119 (18.5) |
| Loss of appetite in the past one month | Yes | 82 (67.8) | 60 (49.6) | 34 (28.1) |
|  | No | 397 (54.5) | 300 (41.2) | 125 (17.2) |
| Stunting | No | 326 (56.4) | 241 (41.7) | 110 (19.0) |
|  | Yes | 153 (56.3) | 119 (43.7) | 49 (18.0) |
| Thinness | No | 422 (55.0) | 314 (40.9) | 141 (18.4) |
|  | Yes | 57 (68 .7) | 46 (55.4) | 18 (21.7) |
| Anemia | No | 306 (54.0) | 225 (39.7) | 92 (16.2) |
|  | Yes | 150 (63.0) | 115 (48.3) | 64 (26.9) |
| De-worming drug past six months | Yes | 109 (57.4) | 82 (43.2) | 28 (14.7) |
|  | No | 370 (56.0) | 278 (42.1) | 131 (19.8) |
| **Individual parent factors** | |  |  |  |
| Mother’s education level | Never entered school | 392 (58.5) | 297 (44.3) | 130 (19.4) |
|  | Read and write only | 47 (58.0) | 33 (40.7) | 17 (21.0) |
|  | Primary and above | 38 (40.0) | 28 (29.5) | 12 (12.6) |
| Father’s education level | Never entered school | 127 (60.2) | 100 (47.4) | 41 (19.4) |
|  | Read and write only | 112 (56) | 78 (39.0) | 39 (19.5) |
|  | Primary and above | 203 (53.7) | 153 (40.5) | 66 (17.5) |
| **Household factor** | |  |  |  |
| Wealth status | Poor | 165 (57.9) | 124 (43.5) | 53 (18.6) |
|  | Middle-class | 165 (56.3) | 121 (41.3) | 53 (18.1) |
|  | Rich | 149 (54.8) | 115 (42.3) | 53 (19.5) |
| Source of drinking water | Unprotected | 213 (58.8) | 150 (41.4) | 90 (24.9) |
|  | Protected | 266 (54.5) | 210 (43.0) | 69 (14.1) |
| Water storage container | Closed container | 440 (55.3) | 331 (41.6) | 149 (18.7) |
|  | Open container | 39 (72.2) | 29 (53.7) | 10 (18.5) |
| Using treated water at household level | Yes | 60 (56) | 42 (39.3) | 19 (17.8) |
|  | No | 419 (56.4) | 318 (42.8) | 140 (18.8) |
| **School factor** | |  |  |  |
| Access to health education on personal hygiene | Yes | 374 (56.4) | 288 (43.4) | 123 (18.6) |
|  | No | 105 (56.2) | 72 (38.5) | 36 (19.3) |
| Absent in the past one  month | Yes | 217 (55.6) | 155 (39.7) | 76 (19.5) |
|  | No | 262 (57) | 205 (44.6) | 83 (18.0) |
| Participates in  school food program | No | 250 (58.7) | 170 (39.9) | 90 (21.1) |
|  | Yes | 229 (54) | 190 (44.8) | 69 (16.3) |

Any helminths: *T. trichiura, A. lumbricoides, Taenia* species, hookworm species, *S. stercoralis, H. nana*
