# Supplementary material for: Helminth infections among rural schoolchildren in Southern Ethiopia: A cross-sectional multilevel and zero-inflated regression model
Source: PLoS Negl Trop Dis. 2020 Dec 22;14(12):e0008002. doi: 10.1371/journal.pntd.0008002 (PMC7755205; doi:10.1371/journal.pntd.0008002)
Supplement: S5 Table — (DOCX) [file pntd.0008002.s007.docx]

S5 Table. The mean, median, SD, and IQR of *T.trichiuria* infection loads in egg per gram of stool among schoolchildren in the Wonago district, Southern Ethiopia, 2017 (n=850)

| **Variables** | | ***T.trichiuria* egg count** | | |
| --- | --- | --- | --- | --- |
|  |  | **Infection free (Epg=0)** | **(Epg > 0)** | **(Epg > 0)** |
| **Individual child factors** | | **n (%)** | **Mean (SD)** | **Median (IQR)** |
| Sex of child | Boys | 273 (57.0) | 138.6 (113.5) | 120 (72-168) |
|  | Girls | 217 (58.5) | 181.8 (406.2) | 120 (72-168) |
| Child age in years | 7-9 | 85 (54.0) | 170.1 (140.6) | 120 (72-192) |
|  | 10-14 | 405 (58.4) | 153.1 (299.1) | 120 (72-168) |
| Finger nail trimmed | Yes | 399 (57.4)) | 145.6 (111.4) | 120 (72-168) |
|  | No | 91 (58.7) | 205.2 (603.2) | 108 (72-144) |
| Dirt on children fingers | Yes | 118 (57.0) | 185.1 (510.6) | 120 (72-168) |
|  | No | 372 (57.8) | 146.6 (114.2) | 120 (72-168) |
| Habit of eating uncooked vegetable | Yes | 110 (52.9) | 146.3 (116.9) | 120 (72-168) |
|  | No | 380 (59.2) | 160.3 (315.8) | 120 (72-168) |
| Loss of appetite in the past one month | Yes | 61 (50.4) | 143.2 (109.5) | 120 (72-168) |
|  | No | 429 (58.8) | 159.1 (297.6) | 120 (72-168) |
| Anemia | No | 342 (60.3) | 154.9 (122.0) | 120 (72-168) |
|  | Yes | 123 (51.7) | 166.6 (456.5) | 120 (72-168) |
| Thinness | No | 453 (59.1) | 157.8 (292.2) | 120 (72-168) |
|  | Yes | 37 (44.6) | 147.3 (104.1) | 120 (72-168) |
| **Individual parent factor** | |  |  |  |
| Mother’s education level | Never entered school | 373 (55.7) | 160.6 (301.2) | 120 (72-168) |
|  | Read and write only | 48 (59.2) | 146.2 (105.9) | 120 (72-168) |
|  | Primary and above | 67 (70.5) | 123.6 (70.0) | 120 (72-168) |
| **Household factor** | |  |  |  |
| Wealth status | Poor | 161 (56.5) | 157.8 (130.7) | 144 (72-192) |
|  | Middle-class | 172 (58.7) | 134.0 (85.6) | 120 (72-168) |
|  | Rich | 157 (57.7) | 178.1 (460.2) | 96 (72-168) |
| Water storage | Closed container | 465 (58.4) | 150.9 (281.7) | 120 (72-168) |
|  | Open container | 25 (46.3) | 226.6 (161.2) | 168 (120-336) |
| **School factor** | |  |  |  |
| Participates in  school food program | No | 256 (60.1) | 135.7 (100.7) | 120 (72-168) |
|  | Yes | 234 (55.2) | 173.9 (362.1) | 120 (72-168) |

EPG: Egg per gram of stool; IQR: Interquartile ranges; SD: Standard deviation
