# Supplementary material for: Helminth infections among rural schoolchildren in Southern Ethiopia: A cross-sectional multilevel and zero-inflated regression model
Source: PLoS Negl Trop Dis. 2020 Dec 22;14(12):e0008002. doi: 10.1371/journal.pntd.0008002 (PMC7755205; doi:10.1371/journal.pntd.0008002)
Supplement: S6 Table — (DOCX) [file pntd.0008002.s008.docx]

S6 Table. The mean, median, SD, and IQR of *A.lumbricoides* infection loads in egg per gram of stool among schoolchildren in the Wonago district, Southern Ethiopia, 2017 (n=850)

| **Variables** | | ***A. lumbricoides* egg count** | | |
| --- | --- | --- | --- | --- |
|  |  | **Infection free (Epg=0)** | **(Epg > 0)** | **(Epg > 0)** |
| **Individual child factors** |  | **n (%)** | **Mean (SD)** | **Median (IQR)** |
| Sex of child | Boys | 383 (80.0) | 321.6 (1213.2) | 120 (96-216) |
|  | Girls | 308 (83.0) | 232.4 (609.3) | 120 (72-204) |
| Child age in years | 7-9 | 135 (86.0) | 124.6 (55.1) | 120 (72-144) |
|  | 10-14 | 556 (80.2) | 311.8 (1086.3) | 120 (72-216) |
| Finger nail trimmed | Yes | 567 (81.6) | 157.8 (107.5) | 120 (72-216) |
|  | No | 124 (80.0) | 839.1 (2272.6) | 120 (96-240) |
| Dirt on children fingers | Yes | 159 (76.8) | 582.5 (1797.7) | 156 (108-216) |
|  | No | 111 (17.3) | 154.9 (112.1) | 120 (72-192) |
| Hand washing with soap after latrine | Always | 80 (78.4) | 204 (142.0) | 132 (72-360) |
|  | Sometimes | 403 (83.8) | 347.8 (1366.2) | 120 (72-192) |
|  | Never | 208 (77.9) | 240.8 (628.2) | 132 (72-216) |
| De-worming drug past six months | Yes | 162 (85.3) | 200 (144.5) | 120 (120-264) |
|  | No | 529 (80.2) | 304.1 (1113.4) | 120 (72-192) |
| Anemia | No | 475 (83.8) | 195.1 (390.5) | 120 (72-204) |
|  | Yes | 174 (73.1) | 227.8 (610.3) | 120 (96-216) |
| **Individual parent factor** |  |  |  |  |
| Mother’s education level | Never entered school | 540 (80.6) | 226.3 (538.4) | 120 (72-216) |
|  | Read and write only | 64 (79.0) | 148.2 (78.8) | 120 (96-216) |
|  | Primary and above | 83 (87.4) | 1314.7 (3557.6) | 120 (96-192) |
| **Household factor** |  |  |  |  |
| Wealth status | Poor | 232 (81.4) | 226.2 (512.7) | 120 (72-216) |
|  | Middle-class | 240 (81.9) | 386 (1538.8) | 120 (72-216) |
|  | Rich | 219 (80.5) | 242.4 (665.4) | 120 (72-192) |
| **School factors** |  |  |  |  |
| Participates in  school food program | No | 336 (78.9) | 145.1 (79.1) | 120 (72-192) |
|  | Yes | 355 (83.7) | 482.4 (1547.6) | 120 (84-228) |

EPG: Egg per gram of stool; IQR: Interquartile ranges; SD: Standard deviation
