# Supplementary material for: Helminth infections among rural schoolchildren in Southern Ethiopia: A cross-sectional multilevel and zero-inflated regression model
Source: PLoS Negl Trop Dis. 2020 Dec 22;14(12):e0008002. doi: 10.1371/journal.pntd.0008002 (PMC7755205; doi:10.1371/journal.pntd.0008002)
Supplement: S7 Table — (DOCX) [file pntd.0008002.s009.docx]

**S7 Table.** Inter-rater agreement for the readings of 85 Kato-Katz microscopic slides

|  | | | **A. lumbricoides** | | | **T. trichiuria** | | | **Taenia spp.** | | | **Hookworm spp.** | | |
| --- | --- | --- | --- | --- | --- | --- | --- | --- | --- | --- | --- | --- | --- | --- |
|  |  |  | + | - | T | + | - | T | + | - | T | + | - | T |
| **First reader** | + | | 34 | 1 | 35 | 41 | 5 | 46 | 27 | 3 | 30 | 22 | 2 | 24 |
|  | - | | 6 | 44 | 50 | 0 | 39 | 39 | 2 | 53 | 55 | 3 | 58 | 61 |
|  | T | | 40 | 45 | 85 | 41 | 44 | 85 | 29 | 56 | 85 | 25 | 60 | 85 |
| **Kappa (95% CI for Kappa)** |  | **0.83 (0.72-0.95)** | | | | **0.88 (0.78-0.98)** | | | **0.87 (0.76-0.98)** | | | **0.86 (0.74-0.98)** | | |

+: positive; -: Negative; T: Total

Kappa values were defined as follows: poor=0.01–0.2; fair=0.21–0.4; moderate=0.41–0.6; good=0.61–0.8; and perfect=0.81–1

Faecal egg counts of the false-positive Kato-Katz thick smears: For A. lumbricoides (10 eggs), for T. trichiuria ( 5, 5, 7, 10, 11 eggs), for Taenia species (5, 5, 11eggs), for Hookworm (5, 6 eggs)

Faecal egg counts of the false-negative Kato-Katz thick smears: For A. lumbricoides (5, 5, 5 9, 9, 7 eggs), for Taenia species (6, 9 eggs), for Hookworm (1, 3, 3 eggs)
