# Supplementary material for: Helminth infections among rural schoolchildren in Southern Ethiopia: A cross-sectional multilevel and zero-inflated regression model
Source: PLoS Negl Trop Dis. 2020 Dec 22;14(12):e0008002. doi: 10.1371/journal.pntd.0008002 (PMC7755205; doi:10.1371/journal.pntd.0008002)
Supplement: S8 Table — (DOCX) [file pntd.0008002.s010.docx]

**S8 Table.** Inter-rater agreement for the readings of 85 FEC microscopic slides.

| **Second reader** | | | | | | | | | | | | | | | | |
| --- | --- | --- | --- | --- | --- | --- | --- | --- | --- | --- | --- | --- | --- | --- | --- | --- |
|  | | **A. lumbricoides** | | | **T. trichiuria** | | | **Taenia spp.** | | | **Hookworm spp.** | | | ***S. stercoralis*** | | |
|  |  | + | - | T | + | - | T | + | - | T | + | - | T | + | - | T |
| **First reader** | + | 37 | 2 | 39 | 24 | 3 | 27 | 19 | 2 | 21 | 7 | 1 | 8 | 10 | 2 | 12 |
|  | - | 2 | 44 | 46 | 2 | 56 | 58 | 3 | 61 | 64 | 1 | 76 | 77 | 2 | 71 | 73 |
|  | T | 39 | 46 | 85 | 26 | 59 | 85 | 22 | 63 | 85 | 8 | 77 | 85 | 12 | 73 | 85 |
| **Kappa (95% CI for Kappa)** |  | **0.91 (0.82-0.99)** | | | **0.86 (0.75-0.98)** | | | **0.84 (0.71-0.97)** | | | **0.86 (0.67-1.00)** | | | **0.81 (0.62-0.99)** | | |

FEC: Formalin-ether concentration; +: positive; -: Negative; T: Total

Kappa values were defined as follows: poor=0.01–0.2; fair=0.21–0.4; moderate=0.41–0.6; good=0.61–0.8; and perfect=0.81–1
