# Supplementary material for: Helminth infections among rural schoolchildren in Southern Ethiopia: A cross-sectional multilevel and zero-inflated regression model
Source: PLoS Negl Trop Dis. 2020 Dec 22;14(12):e0008002. doi: 10.1371/journal.pntd.0008002 (PMC7755205; doi:10.1371/journal.pntd.0008002)
Supplement: S9 Table — (DOCX) [file pntd.0008002.s011.docx]

**S9 Table**. Bivariate, multilevel, mixed-effect, regression analysis of any intestinal helminthes, *T.trichiuria*, and *A.lumbricoides* among schoolchildren in the Wonago district, Southern Ethiopia, 2017

| **Variables** | | **Crude odds ratio (COR)** | | |
| --- | --- | --- | --- | --- |
|  |  | **Any helminths** | ***T.trichiuria*** | ***A.lumbricoides*** |
| **Individual factor of child** | |  |  |  |
| Sex of child | Boys | 1.0 | 1.0 | 1.0 |
|  | Girls | 0.90 (0.68, 1.19) | 0.94 (0.71, 1.24) | 0.82 (0.57, 1.18) |
| Child age in years | 7-9 | 1.16 (0.81, 1.66) | 1.18 (0.83, 1.69) | 0.62 (0.35, 1.08) |
|  | 10-14 | 1.0 | 1.0 | 1.0 |
| Finger nails trimmed | Yes | 0.79 (0.55, 1.14) | 1.05 (0.73, 1.49) | 0.94 (0.59, 1.49) |
|  | No | 1.0 | 1.0 | 1.0 |
| Dirt on children fingers | Yes | 1.13 (0.82, 1.57) | 1.05 (0.76, 1.44) | 1.43 (0.96, 2.15) |
|  | No | 1.0 | 1.0 | 1.0 |
| Hand washing with soap after latrine | Always | 1.0 | 1.0 | 1.0 |
|  | Sometimes | 0.76 (0.48, 1.18) | 0.74 (0.48, 1.15) | 0.71 (0.40, 1.25) |
|  | Never | 0.81 (0.49, 1.34) | 0.73 (0.45, 1.18) | 1.02 (0.56, 1.87) |
| Hand washing with soap before meal | Yes | 0.69 (0.28, 1.70) | 0.65 (0.27, 1.57) | 0.96 (0.31, 2.98) |
|  | No | 1.0 | 1.0 | 1.0 |
| Eats uncooked vegetables | Yes | 1.26 (0.90, 1.77) | 1.31 (0.94, 1.81) | 0.90 (0.57, 1.42) |
|  | No | 1.0 | 1.0 | 1.0 |
| Loss of appetite in the past one month | Yes | 1.67 (1.08, 2.58)* | 1.44 (0.94, 2.19) | 1.74 (1.04, 2.93)* |
|  | No | 1.0 | 1.0 | 1.0 |
| De-worming drug past six months | Yes | 1.0 | 1.0 | 1.0 |
|  | No | 1.01 (0.71, 1.43) | 0.96 (0.68, 1.36) | 1.37 (0.81, 2.32) |
| Stunting | No | 1.0 | 1.0 | 1.0 |
|  | Yes | 1.02 (0.76, 1.36) | 1.09 (0.82, 1.47) | 0.94 (0.64, 1.38) |
| Thinness | No | 1.0 | 1.0 | 1.0 |
|  | Yes | 1.84 (1.13, 3.00)* | 1.80 (1.14, 2.85)* | 1.31 (0.74, 2.31) |
| Anemia | No | 1.0 | 1.0 | 1.0 |
|  | Yes | 1.51 (1.09, 2.08)* | 1.47 (1.06, 2.04) | 1.82 (1.24, 2.67)* |
| **Individual parent factors** | |  |  |  |
| Mother’s education level | Never entered school | 1.97 (1.24, 3.12)* | 1.91 (1.19, 3.05)* | 1.78 (0.92, 3.45) |
|  | Read and write only | 1.84 (0.97, 3.51) | 1.65 (0.88, 3.09) | 1.71 (0.73, 4.00) |
|  | Primary and above | 1.0 | 1.0 | 1.0 |
| Father’s education level | Never entered school | 1.26 (0.89, 1.79) | 1.33 (0.94, 1.87) | 1.17 (0.74, 1.83) |
|  | Read and write only | 1.02 (0.70, 1.48) | 0.95 (0.66, 1.37) | 1.19 (0.74, 1.94) |
|  | Primary and above | 1.0 | 1.0 | 1.0 |
| **Household factor** | |  |  |  |
| Wealth status | Poor | 1.07 (0.76, 1.51) | 1.05 (0.75, 1.47) | 0.96 (0.62, 1.48) |
|  | Middle-class | 0.98 (0.69, 1.39) | 0.96 (0.68, 1.35) | 0.91 (0.58, 1.42) |
|  | Rich | 1.0 | 1.0 | 1.0 |
| Source of drinking water | Unprotected | 1.13 (0.81, 1.57) | 0.94 (0.71, 1.26) | 2.11 (1.40, 3.18)* |
|  | Protected | 1.0 | 1.0 | 1.0 |
| Water storage container | Closed container | 1.0 | 1.0 | 1.0 |
|  | Open container | 2.12 (1.14, 3.95)* | 1.64 (0.94, 2.86) | 0.96 (0.46, 1.98) |
| Using treated water at household level | Yes | 1.0 | 1.0 | 1.0 |
|  | No | 1.01 (0.67, 1.52) | 1.15 (0.76, 1.75) | 1.16 (0.67, 2.01) |
| **School factor** | |  |  |  |
| Access to health education on personal hygiene | Yes | 0.98 (0.66, 1.46) | 1.22 (0.87, 1.72) | 0.93 (0.56, 1.55) |
|  | No | 1.0 | 1.0 | 1.0 |
| Absent in the past one month | Yes | 1.004 (0.76, 1.33) | 0.82 (0.62, 1.08) | 1.09 (0.76, 1.58) |
|  | No | 1.0 | 1.0 | 1.0 |
| Participates in school food program | No | 1.21 (0.78, 1.86) | 0.82 (0.62, 1.08) | 1.41 (0.85, 2.32) |
|  | Yes | 1.0 | 1.0 | 1.0 |

Any helminths: *T. trichiura, A. lumbricoides, Taenia* species, hookworm species, *S. stercoralis, H.nana*;

COR: Crude odds ratio; *P < .05
