# Supplementary material for: Helminth infections among rural schoolchildren in Southern Ethiopia: A cross-sectional multilevel and zero-inflated regression model
Source: PLoS Negl Trop Dis. 2020 Dec 22;14(12):e0008002. doi: 10.1371/journal.pntd.0008002 (PMC7755205; doi:10.1371/journal.pntd.0008002)
Supplement: S10 Table — (DOCX) [file pntd.0008002.s012.docx]

**S10 Table.** Multivariate, multilevel, mixed-effect, regression analysis of any helminth infections among schoolchildren in the Wonago district, Southern Ethiopia, 2017

| **Variables** | | **Any helminths** | **Adjusted OR (95% CI)** | | | | |
| --- | --- | --- | --- | --- | --- | --- | --- |
|  |  | **Yes (%)** | **Model I** | **Model II** | **Model III** | **Model IV** | **Model V** |
| **Individual child factors** | |  |  |  |  |  |  |
| Sex | Boys | 276 (57.6) | - | 1.0 | 1.0 | 1.0 | 1.0 |
|  | Girls | 203 (54.7) | - | 0.96 (0.72, 1.28) | 0.97 (0.72, 1.30) | 0.98 (0.74, 1.32) | 0.98 (0.74, 1.32) |
| Age in years | 7-9 | 92 (58.6) | - | 1.19 (0.82, 1.74) | 1.17 (0.81, 1.71) | 1.13 (0.77, 1.65) | 1.13 (0.77, 1.65) |
|  | 10-14 | 387 (55.8) | - | 1.0 | 1.0 | 1.0 | 1.0 |
| Fingernails trimmed | Yes | 386 (55.5) | - | 0.85 (0.52, 1.39) | 0.85 (0.58, 1.26) | 0.84 (0.57, 1.24) | 0.84 (0.57, 1.24) |
|  | No | 93 (60) | - | 1.0 | 1.0 | 1.0 | 1.0 |
| Dirt on fingers | Yes | 121 (58.5) | - | 1,0 | - | - | - |
|  | No | 358 (55.7) | - | 1.03 (0.66, 1.60) | - | - | - |
| Handwashing with soap after latrine | Always | 63 (61.8) | - | 1.30 (0.76, 2.24) | 1.31 (0.78, 2.18) | - | - |
|  | Sometimes | 264 (54.9) | - | 1.06 (0.72, 1.56) | 1.09 (0.78, 1.53) | - | - |
|  | Never | 152 (56.9) | - | 1,0 | 1,0 | - | - |
| Handwashing before meals | Yes | 466 (56.2) | - | 0.68 (0.27, 1.72) | 0.69 (0.27, 1.75) | 0.72 (0.28, 1.83) | 0.72 (0.28, 1.83) |
|  | No | 13 (61.9) | - | 1.0 | 1.0 | 1.0 | 1.0 |
| Eats uncooked vegetables | Yes | 125 (60) | - | 1.27 (0.88, 1.82) | 1.31 (0.92, 1.86) | 1.35 (0.95, 1.92) | 1.35 (0.95, 1.92) |
|  | No | 354 (55.1) | - | 1,0 | 1,0 | 1,0 | 1,0 |
| Loss of appetite in past month | Yes | 82 (67.8) | - | 1.77 (1.10, 2.85)* | 1.89 (1.18, 3.04)* | 1.89 (1.16, 3.08)* | 1.89 (1.16, 3.08)* |
|  | No | 397 (54.5) | - | 1.0 | 1.0 | 1.0 | 1.0 |
| Thinness | No | 422 (55.0) | - | 1.0 | 1.0 | 1.0 | 1.0 |
|  | Yes | 57 (68 .7) | - | 1.73 (1.04, 2.88)* | 1.68 (1.01, 2.79)* | 1.73 (1.04, 2.90)* | 1.73 (1.04, 2.90)* |
| Anemia | No | 306 (54) | - | 1.0 | 1.0 | 1.0 | 1.0 |
|  | Yes | 150 (63) | - | 1.52 (1.09, 2.12)* | 1.49 (1.07, 2.07)* | 1.45 (1.04, 2.03)* | 1.45 (1.04, 2.03)* |
| De-worming drug past six months | Yes | 109 (57.4) |  | 0.99 (0.67, 1.47) | - | - | - |
|  | No | 370 (56) |  | 1.0 | - | - | - |
| **Individual parent factors** | |  |  |  |  |  |  |
| Mother’s education level | Never entered school | 392 (58.5) |  | - | 2.07 (1.26, 3.41)** | 2.08 (1.25, 3.47)** | 2.08 (1.25, 3.47)** |
|  | Read and write only | 47 (58) |  | - | 1.97 (0.97, 4.02) | 1.91 (0.92, 3.97) | 1.91 (0.92, 3.97) |
|  | Primary and above | 38 (40) |  | - | 1.0 | 1.0 | 1.0 |

AIC: Akaike information criterion; CI: confidence interval; ICC: intra-cluster correlation; NS: Not significant;

OR: odds ratio; **P<.01, *P<.05

Any helminths: *T. trichiura, A. lumbricoides, Taenia* species, hookworm species, *S. stercoralis, H.nana*

**S10 Table.**  Multivariate, multilevel, mixed-effect, regression analysis of any helminth infections among schoolchildren in the Wonago district, Southern Ethiopia, 2017 (Continued)

| **Variables** | | **Any helminths** | **Adjusted OR (95% CI)** | | | | |
| --- | --- | --- | --- | --- | --- | --- | --- |
|  |  | **Yes (%)** | **Model I** | **Model II** | **Model III** | **Model IV** | **Model V** |
| **Household factors** | |  |  |  |  |  |  |
| Wealth | Poor | 165 (57.9) | - | - | - | 0.99 (0.69, 1.43) | 0.99 (0.69, 1.43) |
|  | Middle | 165 (56.3) | - | - | - | 1.08 (0.74, 1.58) | 1.08 (0.74, 1.58) |
|  | Rich | 149 (54.8) | - | - | - | 1.0 | 1.0 |
| Source of drinking water | Unprotected | 213 (58.8) |  | - | - | 0.98 (0.67, 1.44) | 1.02 (0.69, 1.49) |
|  | Protected | 266 (54.5) | - | - | - | 1.0 | 1.0 |
| Water storage container | Closed container | 440 (55.3) | - | - | - | 1.0 | 1.0 |
|  | Open container | 39 (72.2) | - | - | - | 2.06 (1.07, 3.99)* | 2.06 (1.07, 3.99)* |
| Using treated water at home | Yes | 60 (56.0) | - | - | - | 1.07 (0.68, 1.68) | 1.07 (0.68, 1.68) |
|  | No | 419 (56.4) | - | - | - | 1.0 | 1.0 |
| **School factor** | |  |  |  |  |  |  |
| Participates in  school food program | No | 250 (58.7) | - | - | - | - | 1.0 |
|  | Yes | 229 (54.0) | - | - | - | - | 0.98 (0.66, 1.46) |
| **Variation and model fitness** | |  |  |  |  |  |  |
| Variance | School level |  | 0.04 | 0.023 | 0.002 | 0.003 | 0.003 |
|  | Class level |  | NS | NS | NS | NS | NS |
| ICC | School |  | 1.2% | 1% | 0.1% | 0.1% | 0.1% |
|  | Class |  | 1.2% | 1% | 0.1% | 0.1% | 0.1% |
| **Model fitness** | |  |  |  |  |  |  |
| -2log likelihood | |  | 1160 | 1076 | 1062 | 1057 | 1056 |
| AIC | |  | 1165 | 1104 | 1089 | 1091 | 1093 |

AIC: Akaike information criterion; CI: confidence interval; ICC: intra-cluster correlation; NS: Not significant;

OR: odds ratio; **P<.01, *P<.05

Any helminths: *T. trichiura, A. lumbricoides, Taenia* species, hookworm species, *S. stercoralis, H.nana*
