# Supplementary material for: Helminth infections among rural schoolchildren in Southern Ethiopia: A cross-sectional multilevel and zero-inflated regression model
Source: PLoS Negl Trop Dis. 2020 Dec 22;14(12):e0008002. doi: 10.1371/journal.pntd.0008002 (PMC7755205; doi:10.1371/journal.pntd.0008002)
Supplement: S11 Table — (DOCX) [file pntd.0008002.s013.docx]

**S11 Table.** Multivariate, multilevel, mixed-effect, logistic regression analysis of *T.trichiuria* infection among schoolchildren in the Wonago district, Southern Ethiopia, 2017

| **Variables** | | ***T.trichiuria*** | **Adjusted OR (95% CI)** | | | | |
| --- | --- | --- | --- | --- | --- | --- | --- |
| **Individual child factors** | | **Yes (n (%)** | **Model I** | **Model II** | **Model III** | **Model IV** | **Model V** |
| Sex of child | Boys | 206 (43.0) | - | 1.0 | 1.0 | 1.0 | 1.0 |
|  | Girls | 154 (41.5) | - | 1.02 (0.76, 1.35) | 1.02 (0.76, 1.35) | 1.02 (0.76, 1.36) | 1.02 (0.76, 1.36) |
| Child age in years | 7-9 | 72 (46.0) | - | 1.0 | 1.0 | 1.0 | 1.0 |
|  | 10-14 | 288 (41.6) | - | 0.78 (0.53, 1.15) | 0.78 (0.53, 1.16) | 0.81 (0.55, 1.21) | 0.81 (0.55, 1.17) |
| Habit of eating uncooked vegetable | Yes | 98 (47.1) | - | 1.31 (0.92, 1.85) | 1.34 (0.94, 1.90) | 1.34 (0.94, 1.91) | 1.32 (0.94, 1.86) |
|  | No | 262 (40.8) | - | 1.0 | 1.0 | 1.0 | 1.0 |
| Loss of appetite in the past one month | Yes | 60 (49.6) | - | 1.59 (1.03, 2.47)* | 1.59 (1.03, 2.47)* | 1.58 (1.01, 2.47)* | 1.76 (1.15, 2.71)* |
|  | No | 300 (41.2) | - | 1.0 | 1.0 | 1.0 | 1.0 |
| Thinness | No | 314 (40.9) |  | 1.0 | 1.0 | 1.0 | 1.0 |
|  | Yes | 46 (55.4) |  | 1.71 (1.07, 2.75)* | 1.69 (1.05, 2.72)* | 1.73 (1.07, 2.79)* | 1.73 (1.07, 2.78)* |
| Anemia | No | 225 (39.7) |  | 1.0 | 1.0 | 1.0 | 1.0 |
|  | Yes | 115 (48.3) |  | 1.47 (1.06, 2.75)* | 1.46 (1.05, 2.03)* | 1.48 (1.07, 2.07)* | 1.53 (1.11, 2.12)** |
| **Individual parent factors** | |  |  |  |  |  |  |
| Mother’s education level | Never entered school | 297 (44.3) | - | - | 1.81 (1.09, 2.97)* | 1.84 (1.10, 2.94)* | 1.94 (1.18, 3.19)** |
|  | Read and write only | 33 (40.7) | - | - | 1.42 (0.72, 2.84) | 1.46 (0.73, 2.94) | 1.54 (0.78, 3.04) |
|  | Primary and above | 28 (29.5) | - | - | 1.0 | 1.0 | 1.0 |
| **Household factor** | |  |  |  |  |  |  |
| Wealth status | Poor | 124 (43.5) | - | - | - | 0.95 (0.66, 1.36) | 0.98 (0.69, 1.41) |
|  | Middle | 121 (41.3) | - | - | - | 0.98 (0.68, 1.42) | 1.06 (0.74, 1.52) |
|  | Rich | 115 (42.3) | - | - | - | 1.0 | 1.0 |
| Family size | 1-4 | 39 (50.6) |  |  |  | 1.0 | 1.0 |
|  | ≥5 | 321 (41.5) |  |  |  | 0.72 (0.44, 1.18) | 0.72 (0.44, 1.19) |
| Water storage | Closed container | 331 (41.6) |  |  |  | 1.56 (0.85, 2.87) | 1.62 (0.89, 2.96) |
|  | Open container | 29 (53.7) |  |  |  |  | 1.0 |
| **School factor** | |  |  | - |  |  |  |
| Participates in  school food program | No | 170 (39.9) | - | - | - | - | 1.0 |
|  | Yes | 190 (44.8) | - | - | - | - | 1.55 (1.13, 2.12)** |
| **Variation and model fitness** | |  |  |  |  |  |  |
| Variance | School level |  | NS | 0.018 | 0.016 | NS | NS |
|  | Class level |  | 0.016 | NS | NS | 0.013 | NS |
| ICC | School |  | NS | 0.5% | 0.5% | 0.6% | NS |
|  | Class |  | 0.5% | 0.7% | 0.7% | 0.9% | NS |
| Model fitness | |  |  |  |  |  |  |
| -2log likelihood | |  | 1158 | 1078 | 1066 | 1062 | 1054 |
| AIC | |  | 1162 | 1096 | 1088 | 1092 | 1083 |

AIC: Akaike information criterion; CI: confidence interval; ICC: intra-cluster correlation; NS: Not significant; OR: odds ratio; **P<.01, *P<.05
