# Supplementary material for: Helminth infections among rural schoolchildren in Southern Ethiopia: A cross-sectional multilevel and zero-inflated regression model
Source: PLoS Negl Trop Dis. 2020 Dec 22;14(12):e0008002. doi: 10.1371/journal.pntd.0008002 (PMC7755205; doi:10.1371/journal.pntd.0008002)
Supplement: S12 Table — (DOCX) [file pntd.0008002.s014.docx]

**S12 Table.** Multivariate, multilevel, mixed-effect, logistic regression analysis of *A.lumbricoides* infection among schoolchildren in the Wonago district, Southern Ethiopia, 2017

| **Variables** | | ***A. lumbricoides*** | **Adjusted OR (95% CI)** | | | | |
| --- | --- | --- | --- | --- | --- | --- | --- |
| **Individual child factors** | | **Yes (n (%)** | **Model I** | **Model II** | **Model III** | **Model IV** | **Model V** |
| Sex of child | Boys | 96 (20.0) | - | 1.0 | 1.0 | 1.0 | 1.0 |
|  | Girls | 63 (17.0) | - | 0.86 (0.59, 1.25) | 0.88 (0.61, 1.28) | 0.87 (0.60, 1.27) | 0.89 (0.60, 1.28) |
| Child age in years | 7-9 | 22 (14.0) | - | 1.64 (0.93, 2.88) | 1.62 (0.93, 2.85) | 1.52 (0.88, 2.72) | 1.52 (0.87, 2.68) |
|  | 10-14 | 137 (19.8) | - | 1.0 | 1.0 | 1.0 | 1.0 |
| Finger nail trimmed | Yes | 128 (18.4) | - | 1.52 (0.84, 2.75) | 1.47 (0.81, 2.66) | 1.41 (0.78, 2.56) | 1.41 (0.78, 2.56) |
|  | No | 31 (20.0) | - | 1.0 | 1.0 | 1.0 | 1.0 |
| Dirt on children fingers | Yes | 48 (23.2) | - | 1.59 (0.93, 2.70) | 1.60 (0.94, 2.73) | 1.53 (0.89, 2.61) | 1.54 (0.90, 2.63) |
|  | No | 532 (82.7) | - | 1.0 | 1.0 | 1.0 | 1.0 |
| Hand washing with soap after latrine | Always | 22 (21.6) | - | 1.27 (0.67, 2.41) | 1.29 (0.68, 2.47) | 1.55 (0.80, 3.01) | 1.45 (0.74, 2.85) |
|  | Sometimes | 78 (16.2) | - | 0.74 (0.46, 1.18) | 0.74 (0.46, 1.18) | 0.94 (0.57, 1.55) | 0.88 (0.53, 1.49) |
|  | Never | 59 (22.1) | - | 1.0 | 1.0 | 1.0 | 1.0 |
| Loss of appetite in the past one month | Yes | 34 (28.1) | - | 1.61 (0.95, 2.73) | 1.58 (0.93, 2.69) | 1.41 (0.82, 2.43) | 1.45 (0.84, 2.51) |
|  | No | 125 (17.2) | - | 1.0 | 1.0 | 1.0 | 1.0 |
| Anemia | No | 92 (16.2) | - | 1.0 | 1.0 | 1.0 | 1.0 |
|  | Yes | 64 (26.9) | - | 1.96 (1.32, 2.92)** | 1.95 (1.31, 2.90)** | 1.89 (1.27, 2.82)** | 1.92 (1.29, 2.88)** |
| De-worming drug past six months | Yes | 28 (14.7) |  | 0.65 (0.38, 1.11) | 0.62 (0.36, 1.06) | 0.58 (0.33, .99) | 0.57 (0.33, 0.98)* |
|  | No | 131 (19.8) | - | 1.0 | 1.0 | 1.0 | 1.0 |
| **Individual parent factors** | |  |  |  |  |  |  |
| Mother’s education level | Never entered school | 130 (19.4) | - | - | 1.80 (0.90, 3.60) | 1.67 (0.83, 3.37) | 1.68 (0.84, 3.39) |
|  | Read and write only | 17 (21.0) | - | - | 2.01 (0.83, 4.89) | 1.73 (0.70, 4.24) | 1.71 (0.69, 4.19) |
|  | Primary and above | 12 (12.6) | - | - | 1.0 | 1.0 | 1.0 |
| **Household factor** | |  |  |  |  |  |  |
| Wealth status | Poor | 53 (18.6) | - | - | - | 0.86 (0.55, 1.37) | 0.87 (0.55, 1.38) |
|  | Middle | 53 (18.1) | - | - | - | 0.81 (0.51, 1.30) | 0.83 (0.52, 1.33) |
|  | Rich | 53 (19.5) | - | - | - | 1.0 | 1.0 |
| Source of drinking water | Unprotected | 90 (24.9) | - | - | - | 1.0 | 1.0 |
|  | Protected | 69 (14.1) | - | - | - | 0.51 (0.32, 0.81)** | 0.46 (0.28, 0.77)** |
| **School factor** | |  |  | - |  |  |  |
| Participates in school food program | No | 90 (21.1) | - | - | - | - | 1.0 |
|  | Yes | 69 (16.3) | - | - | - | - | 1.29 (0.71, 2.34) |
| **Variation and model fitness** | |  |  |  |  |  |  |
| Variance | School level |  | NS | NS | NS | NS | NS |
|  | Class level |  | 0.22 | 0.12 | 0.11 | 0.14 | 0.11 |
| ICC | School |  | NS | NS | NS | NS | NS |
|  | Class |  | 6.2% | 3.5% | 3.3% | 4.1% | 3.2% |
| **Model fitness** | |  |  |  |  |  |  |
| -2log likelihood | |  | 810 | 755 | 748 | 738 | 740 |
| AIC | |  | 814 | 777 | 776 | 775 | 774 |

AIC: Akaike information criterion; CI: confidence interval; ICC: intra-cluster correlation; NS: Not significant; OR: odds ratio; **P<.01, *P<.05
