# Supplementary material for: Helminth infections among rural schoolchildren in Southern Ethiopia: A cross-sectional multilevel and zero-inflated regression model
Source: PLoS Negl Trop Dis. 2020 Dec 22;14(12):e0008002. doi: 10.1371/journal.pntd.0008002 (PMC7755205; doi:10.1371/journal.pntd.0008002)
Supplement: S13 Table — (DOCX) [file pntd.0008002.s015.docx]

**S13 Table.** Model validation for *T.trichiuria* and *A.lumbricoides* egg count model.

| **Model fitness** | ***T.trichiuria* model** | |
| --- | --- | --- |
|  | **ZIP** | **ZINB** |
| -2 Log-likelihood (Deviance) | 43126 | 4840 |
| Likelihood ratio test | - | 38000 (P < 0.001) |
| Vuong test | - | 17.5 (P < 0.001) |
| Akaike information criterion (AIC) | 43186 | 4902 |
|  | ***A.lumbricoides* model** | |
| -2 Log-likelihood (Deviance) | 23788 | 2436 |
| Likelihood ratio test | - | 21000 (P<.001) |
| Vuong test | - | 9.4 (P<.001) |
| Akaike information criterion (AIC) | 23845 | 2494 |

ZINB: Zero-inflated negative binomial regression; ZIP: Zero-inflated Poisson regression
